# Zimbro (*Juniperus communis* L.) as a Promising Source of Bioactive Compounds and Biomedical Activities: A Review on Recent Trends

**DOI:** 10.3390/ijms23063197

**Published:** 2022-03-16

**Authors:** Ana C. Gonçalves, José David Flores-Félix, Paula Coutinho, Gilberto Alves, Luís R. Silva

**Affiliations:** 1 CICS-UBI—Health Sciences Research Centre, University of Beira Interior, 6201-506 Covilhã, Portugal; anacarolinagoncalves@sapo.pt (A.C.G.); jdflores@usal.es (J.D.F.-F.); coutinho@ipg.pt (P.C.); gilberto@fcsaude.ubi.pt (G.A.); 2 CPIRN-UDI/IPG—Center of Potential and Innovation of Natural Resources, Research Unit for Inland Development (UDI), Polytechnic Institute of Guarda, 6300-559 Guarda, Portugal

**Keywords:** bioactive compounds, phenolic compounds, essential oils, biological potential, in vitro studies, in vivo studies, *Juniperus communis* L.

## Abstract

Plant-derived products and their extracted compounds have been used in folk medicine since early times. Zimbro or common juniper (*Juniperus communis*) is traditionally used to treat renal suppression, acute and chronic cystitis, bladder catarrh, albuminuria, leucorrhea, and amenorrhea. These uses are mainly attributed to its bioactive composition, which is very rich in phenolics, terpenoids, organic acids, alkaloids, and volatile compounds. In the last few years, several studies have analyzed the huge potential of this evergreen shrub, describing a wide range of activities with relevance in different biomedical discipline areas, namely antimicrobial potential against human pathogens and foodborne microorganisms, notorious antioxidant and anti-inflammatory activities, antidiabetic, antihypercholesterolemic and antihyperlipidemic effects, and neuroprotective action, as well as antiproliferative ability against cancer cells and the ability to activate inductive hepato-, renal- and gastroprotective mechanisms. Owing to these promising activities, extracts and bioactive compounds of juniper could be useful for the development of new pharmacological applications in the treatment of several acute and chronic human diseases.

## 1. Introduction

Natural products have an important role in the research and development of new drugs. People have always extracted natural products from several natural sources, such as marine organisms, microorganisms, animals, and medicinal plants [1]. The main extracts from natural products come from medicinal plants. Plant-derived products and compounds have been used worldwide since ancient times in folk medicine as remedies for several diseases, such as tinctures, teas, poultices, maintaining high prevalence in public health [1,2,3,4]. Advances in clinical research and quality control have shown a greater value of herbal medicine in the treatment and overcoming of many diseases. Recent works report promising potential regarding the use of plants in the treatment and/or prevention of several hard-to-cure diseases, such as atherosclerosis [5,6], cancer [1,2,3,7,8], cardiovascular diseases [9,10,11,12], diabetes [8,13,14], and neurological disorders [4,15,16], among others.

The genus *Juniperus* includes roughly 68 species and 36 varieties and belongs to the Cupressaceae family [17]. The plant *Juniperus communis* L., named “zimbro” in Portugal, is a shrub or small evergreen tree; a perennial and long-lived coniferous, woody pioneer and colonizing plant, adapted to low nutrient availability in soil and having one the widest distribution ranges among the different plant species [18]. Its population is spread globally, being the only *Juniperus* species found in both hemispheres, with reports of this plant in Arctic regions of Asia and North America. In Europe, the largest population is found in some parts of the Alps, Scandinavia, Poland, northwest European lowlands, and Mediterranean mountain regions [19,20]. A significant population of “zimbro” is found in the Natural Park of Serra da Estrela, Portugal, where the var. *alpina* is mainly found at higher altitudes and the var. *hemisphaerica* at lower altitudes [21]. The wide geographical distribution is the principal reason for the remarkable variation in the morphological characteristics and secondary metabolites’ chemical composition [17].

*J. communis* has been used traditionally in folk medicine for renal suppression, acute and chronic cystitis, catarrh of the bladder, albuminuria, leucorrhea, and amenorrhea [22]. Indeed, this plant presents carminative, diuretic, emmenagogue, digestive, anti-inflammatory [23,24,25], antifungal [26,27], antibacterial [26,28], analgesic [29], hepatoprotective [30], antidiabetic and antihyperlipidemic [31], antioxidant [32], antihypercholesterolemic [33], and anticataleptic effects, and the ability to act as a neuroprotective agent against Parkinson’s disease [19,22,34]. Particularly, their berries can be used as antiseptic, stimulant, and styptic agents, and in the treatment of migraine, infantile tuberculosis, rheumatic arthritis, gout and painful swellings, chronic Bright’s disease, piles, and nephrotic dropsy in children [19]. Additionally, it is known that Native Americans used berries as a female contraceptive, anorexigenic agent, and in the treatment of diabetes [35]. Their essential oils are aromatic, possessing a light fruity fragrance which is considered psychologically uplifting [35]. Concerning their dried berries, they are largely used to flavor meat, soups, sauces, stews, stuffing, pickled foods, gin, liquors, bitters, Swedish beer, and borovička (a Slovak national alcoholic beverage similar to gin), being firstly crushed or grounded to release their flavor before being added to dishes [17,35].

On the other hand, *J. communis* seeds are too bitter due to their astringency. They are rarely consumed raw, usually being dried for use as a culinary component in different parts of the world. Together with juniper berries, they are commonly burnt in temples during religious ceremonies to purify the ambient air [36].

Relatively to their composition, “zimbro” plant parts are mostly composed of sugars, resins, organic acids, alkaloids, terpenic acids, leucoanthocyanins and flavonoids, gums, lignins, and wax. Their aromatic oils are rich in hydrocarbons of monoterpenes (*α*-pinene, *β*-pinene, sabinene, and myrcene), diterpenes, and sesquiterpenes [17,19,23,32,35,37]. All of them contribute to the health-promoting properties shown by this plant [34,38,39,40].

This review will outline the experimental technical progress of broad interest in *J. communis* vegetal part’s phytochemical composition and their importance in the treatment of diseases, summarizing the main effects of their active natural compounds in medical therapy.

## 2. Scientific Classification

*J. communis* species belongs to the Pinopsida class, Pinophyta division, Pinales order, Cupressaceae family, and *Juniperus* genus. Its binominal name is *J. communis* L.

The *Juniperus* genus is one of the most diverse within the conifers, being placed in the *Cupressaceae* family and including more than 60 species. It presents cosmopolitan distribution with a great capacity to develop in xerophytic and salinity conditions, although it is easier to find them in high-rainfall regions [41,42]. They are dioecious trees, producing seeds every 2 or 3 years that may have a globose or spherical morphology and be dispersed by zoocoria (e.g., frugivorous birds and small mammals), which allows them to colonize new territories quickly. In addition, they can be differentiated taxonomically into three well-differentiated sections, which represent different degrees of evolution within the genus, according to genetic analyses [41,43]. The *Caryocedrus* section, which is considered the most ancestral from an evolutionary point of view, is limited to areas of the Peloponnese, Anatolia, and Asia Minor and is only represented by the species *J. drupacea*, with acicular leaves, an anchor point to the stem, and woody cones [44]. The *Juniperus* (=*Oxycedrus*) section has a Holarctic distribution reaching the Mediterranean; it is represented by 14 species with acicular leaves, an anchoring point to the stem, and resiniferous cones. The third section, the *Sabina* section, is mainly found in the northern hemisphere and mountainous areas of the African continent. However, it also has some type of resin, and is distinguished from the other ones since it has decurrent needle-like or scale-shaped leaves and juicy cones [45]. According to these characteristics and with the fossil record, it is thought that the diffusion point of this genus occurred in the eastern Mediterranean region, first colonizing the northern regions of the Eurasian continent, and from there passing to the American continent at least 25 My ago [43].

**Figure 1:**
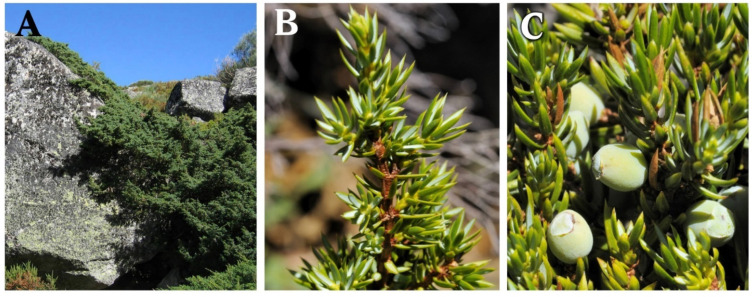
*Juniperus communis* (**A**) main view of the plant growing in Serra da Estrela, (**B**) detail of leaves, and (**C**) details of berries. Images under Creative Commons licence, authorship: João Domingues Almeida and Paulo Ventura Araújo from www.flora-on.pt, accessed on 10 March 2022.

All juniper species stand out for their high content of essential oils and phenolic compounds and are largely included in the traditional medicine of different cultures throughout the planet, exhibiting a wide range of biological activities and industrial applications [46]. Among them, it is worth highlighting the “zimbro” (*J. communis*) plant, since it shows the widest distribution, being practically circumboreal [43,47]. Another remarkable characteristic of this species is the ecological plasticity supported by great genetic variability, which translates into a substantially high number of varieties with phenotypes ranging from medium-sized trees (3–4 m high) to small creeping shrubs (Figure 1) [41,43]. The populations of the Iberian Peninsula are very diverse, and due to their position, their distribution is relegated to mountainous and more humid areas with a territorial occupation in islands, thus scaping from the thermophilic and xeric character of the nonmountainous lands of the Peninsula [44,47]. Indeed, it is believed that many years ago, this territory acted as a glacial refuge for many varieties that are currently found further north; even so, the populations of var. *hemisphaerica* show a high degree of genetic uniqueness, while the var. *alpina* (also known as var. *nana*) is mainly distributed in the upper areas of the mountains of the Iberian Peninsula, such as the Serra da Estrela mountains. These mountains are located in the middle interior of mainland Portugal and display an oromediterranean climatic island, being an isolated population from other populations of the Central System mountains or Cantabric System mountains [21,48].

As well as other plants, *J. communis* also receives popular names, both in our own and in foreign languages. For example, Havusa or Matsyagandha (Sanskrit); Arar, Abahal oe Habbul (Assamese); Hayusha (Bengali); juniper berry, or common juniper (English); Palash (Gujrati); havuber or havubair (Hindi); zimbro (Portuguese); padma beeja (Kannada); hosh (Marathi); havulber (Punjabi); hapusha, abhal or arar (Urdu) [21,23].

For curiosity, and despite this plant not having a strong presence in ancient mythology, it is considered a symbol of fertility in Syria. On the other hand, in the Old Testament, it is described that the juniper has an angelic presence, which sheltered the prophet Elijah from Queen Jezebel’s pursuit. Moreover, a posteriorly biblical tale described that during their flight to Egypt, the infant Jesus and his parents used juniper to hide from King Herod’s soldiers [49].

## 3. Phytochemical Composition of *Juniperus communis* L.

**Figure 2:**
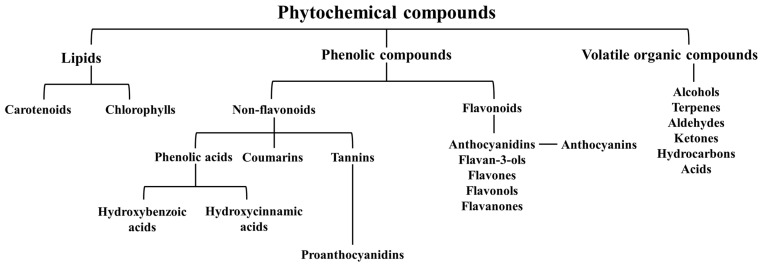
Main phytochemicals found in *Juniperus communis* L. (adapted from Fátima et al. [55]).

As mentioned before, *J. communis* L. species are composed of a myriad of constituents, including nonessential substances, i.e., phytochemicals [50]. These compounds are secondary metabolites produced by plants to promote their normal cellular metabolism and offer protection against biotic and abiotic factors, and consequent oxidative injury [51]. Additionally, they are considered the key contributors to the organoleptic characteristics (e.g., aroma and color) and health benefits exhibited by plants [52]. They can be divided into five major categories (Figure 2). Although the plants’ genotype mainly influences their quantitative and qualitative composition, their levels also depend on the plant’s age, ripeness degree, cultivation techniques, geographical location, and meteorological conditions [53,54].

### 3.1. Carotenoids and Chlorophylls

Although no studies have specifically reported the chlorophyll content of *J. communis* L. species, Rabska and colleagues [56] analyzed their total levels in fertilized and nonfertilized in both genders of this plant in autumn and winter (species not specified). The obtained data revealed nonfertilized plants had a lower concentration of total chlorophyll content than the fertilized ones (mean values of 5.0 versus (vs.) 7.4 mg/g in autumn and 3.6 vs. 4.8 mg/g in winter, respectively), and also lower amounts of total carotenoids (mean values of 0.64 and 0.95 mg/g for female and male, respectively, in autumn, and scores of 0.87 against 1.2 mg/g in winter). Focusing on gender, they observed that female plants had lower amounts of total chlorophyll compounds (values of 2.9 and 4.5 mg/g for female plants in autumn and winter, respectively, and 3.7 and 5.2 mg/g in autumn and winter, respectively, for the male ones) and carotenoid levels (values around 0.90 mg/g for female plants and around 1.0 mg/g for male, in autumn and winter, respectively). Without surprises, and regarding all the comparisons made, the authors also concluded that male and fertilized plants presented the highest levels of total chlorophyll and carotenoids (mean values of 4.3 and 1.3 mg/g, respectively).

This subclass of phytochemicals, highlighting carotenoids, possesses notable antioxidant potential and the ability to easily activate metabolic detoxification pathways, reducing the risk of appearance of several chronic and degenerative disorders [51,56].

### 3.2. Phenolic Compounds

Phenolics are the most predominant phytochemicals present in nature, and to date, about 10,000 different structures are currently described [57]. They are usually classified in (i) nonflavonoids and (ii) flavonoids [58]. The first ones can be further categorized into phenolic acids, including hydroxycinnamic and hydroxybenzoic acids, or in coumarins, lignans, or stilbenes [57]. On the other hand, flavonoids can be subdivided into isoflavones, coumestans, anthocyanidins, flavan-3-ols, flavanones, flavanonols (also called dihydroflavonols), flavones, or flavonols (Figure 2), depending on their structure [54]. This one comprises, at least, one phenol ring attached to one or more hydroxyl groups, and it is not only the main one responsible for dividing phenolics into different subclasses but also for conferring them a notable capacity to easily scavenge free radicals and reactive species; and to chelate metals, and in this way, counteract oxidative stress, diminish proinflammatory markers, and contribute to a healthy life state [57,59].

Focusing on phenolics found in *J. communis* L. species (Table 1), their levels depend on genotype, plant part, origin, age, gender, and solvent used to extract phenolics and perform the studies, but in a general way, they increase with latitude and plant age [56,60,61]. Additionally, male leaves and berries often present higher content in phenolic compounds than female ones [62].

Furthermore, and knowing the current interest in the biological potential of this plant, Brodowska et al. [63] conducted a study where they subjected *J. communis* (var. *communis*) L. berries to different ozone concentrations and time treatments. They verified that the treatment with ozone concentrations of 100 and 130 g/m^3^ for 30 min almost duplicated the phenolic content (15.47 and 12.91 mg catechin equivalent per g dry weight (dw), when compared to control (9.81 mg catechin equivalent per g dw), which consequently enhanced their antioxidant capacities positively.

**Figure 3:**
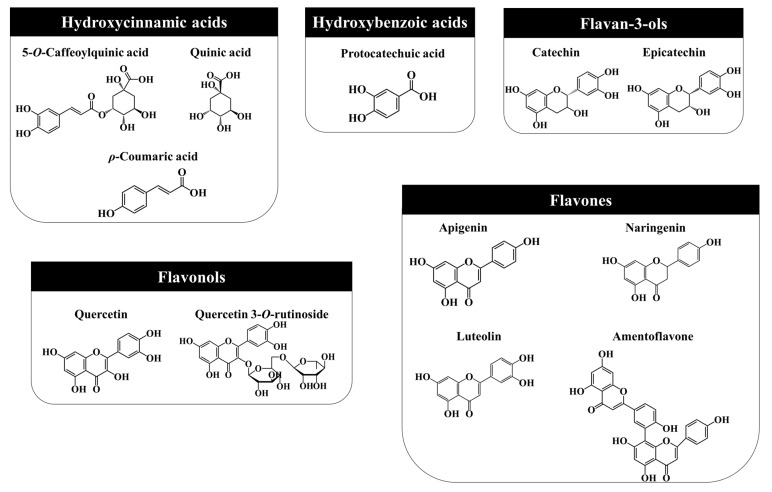
Main phenolic compounds found in *Juniperus communis* L. vegetal parts.

Generally, the majority of phenolics reported in *J. communis* L. plant parts include 5-*O*-caffeoylquinic and quinic acids, catechin, epicatechin, amentoflavone, quercetin, luteolin, apigenin, and naringenin and their derivatives (Figure 3).

**Table 1:** Total phenolic, flavonoid, anthocyanin, and tannin content of different *Juniperus communis* L. plant parts extracts.

	Gender	Origin	Extract	Total Phenolic Compounds ^a^	Total Flavonoid Content ^b^	Total Anthocyanin Content ^c^	Total Tannin Content ^b^	References
Leaves								
*J. communis* (var. *alpina*)	n.s.	Serra Da Estrela, Portugal	Methanolic (100%, *v/v*)	155.60			60.40	[64]
*J. communis* (var. *alpina*)	n.s.	Yozgat, Turkey	Hydroethanolic (80% ethanol, *v/v)*	4.36	7.05			[61]
*J. communis* (var. *alpina*)	n.s.	Yozgat, Turkey	Aqueous	169.27	24.30			
*J. communis* (var. *communis)*	Female	Rhodopes, Bulgaria	Methanolic (80% methanol, *v/v*)	132.00				[60]
*J. communis* (var. *communis)*	Female	Mountain Ozren, near Sarajevo, Bosnia, and Herzegovina	Methanolic (80% methanol, *v/v*)	390.89	40.22 *			[62]
*J. communis* (var. *communis)*	Male	Mountain Ozren, near Sarajevo, Bosnia, and Herzegovina	Methanolic (80% methanol, *v/v*)	544.09	48.06 *			
*J. communis* (var. *communis*)	n.s.	Nainital, India	Hydroethanolic (70% ethanol, *v/v*)	238.78				[65]
*J. communis* (var. *communis*)	n.s.	Nainital, India	Hexane	189.65				
*J. communis* (var. *communis*)	n.s.	Nainital, India	Ethyl acetate	315.33				
*J. communis* (var. *communis*)	n.s.	Nainital, India	Aqueous	205.33				
*J. communis* (var. *oblonga pendula*)	Male	North Carolina, USA	Methanolic (80% methanol, *v/v*)	91.00				[60]
*J. communis* (var. *saxatiles*)	n.s.	Turkey	Hydroethanolic (80% ethanol, *v/v*)	212.10				[66]
**Berries**								
*J. communis* (var. *alpina*)	n.s.	Yozgat, Turkey	Hydroethanolic (80% ethanol, *v/v)*	Ripe berry: 11.92 Unripe berry: 130.92	Ripe berry: 2.56 Unripe berry: 17.57			[61]
*J. communis* (var. *alpina*)	n.s.	Yozgat, Turkey	Aqueous	Ripe berry: 4.36	Ripe berry: 7.05			
*J. communis* (var. *communis*)		North-East Slovakia	Hydroethanolic (70% ethanol, *v/v*)	Ripe berry: 6.87–42.23				[67]
*J. communis* (var. *communis*)	n.s.	Melbourne, Australia	Hydroethanolic (30% ethanol, *v/v*)	Ripe berry: 9.08	Ripe berry: 2.25		**Ripe berry**: 3.48 *	[68]
*J. communis* (var. *communis*)	n.s.	Quebec, Canada	Hydroethanolic (80% ethanol, *v/v*)	Ripe berry: 99.20 ^b^		Ripe berry: 0.47		[69]
*J. communis* (var. *communis*)	n.s.	Serra Da Estrela, Portugal	Methanolic (100%, *v/v*)	Ripe berry: 44.70				[64]
*J. communis* (var. *communis*)	n.s.	Ağrı, Turkey	Methanolic (100%, *v/v*)	Ripe berry: 59.17				[70]
*J. communis* (var. n.s.)	n.s.	Pitesti hills, Romania	Hydroethanolic (50% ethanol, *v/v*)	Ripe berry: 0.19	Ripe berry: 51.09 ^d^			[40]
*J. communis* (*var. saxatilis*)	n.s.	Yozgat, Turkey	Hydroethanolic (80% ethanol, *v/v)*	Ripe berry: 21.00				[66]
*J. communis* (var. *saxatilis*)	n.s.	Ankara, Turkey	Methanolic (100%, *v/v*)	Ripe berry: 17.64				[70]
*J. communis* (n.s.)	n.s.	Šara mountain in south Serbia	Chloroformic	189.82	27.11 ^d^			[50]
*J. communis* (var. n.s.)	n.s.	Šara mountain in south Serbia	Ethanolic	189.82	42.85 ^d^			
*J. communis* (var. n.s.)	n.s.	Šara mountain in south Serbia	Ethyl acetate	144.21	38.40 ^d^			
**Stems**								
*J. communis* (var. *alpina*)	n.s.	Serra Da Estrela, Portugal	Methanolic (100%, *v/v*)	221.30			79.30	[64]

n.s.: not specified; ^a^ mg equivalent of gallic acid (GAE) per g dry weight (dw); ^b^ mg quercetin equivalents per g dw; ^c^ mg cyanidin 3-glucoside equivalents per g dw; ^d^ mg quercetin 3-*O*-rutinoside equivalents per g dw; * mg of catechin equivalents per g dw.

#### 3.2.1. Hydroxycinnamic Acids

The presence of hydroxycinnamic acids on *J. communis* L. leaves is already known, and their levels mainly vary depending on genotype and local origin [71]. For example, 5-*O*-chlorogenic acid was found in ethanolic extracts (50% ethanol, *v/v*) of common *J. communis* L. (var n.s.) from Romania at mean values of 6.8 mg/g of dw [40]. On the other hand, quinic, cinnamic, *ρ*-coumaric, ferulic, and caffeic acids were detected in methanolic extracts (80% methanol, *v/v*) of var. *saxatilis* at concentrations of 11.1, 0.097, 0.088, 0.053, and <0.002 mg/g of dw, respectively [71]. Higher amounts of *ρ*-coumaric acid were found in methanolic extracts (80% methanol, *v/v*) of var. *laxa* (c.a. 5.95 mg/g of dw) [60]. 

Concerning *J. communis* L. berries, the presence of isoferulic acid, verbascoside, *ρ*-coumaric, cinnamic, quinic, and sinapic acid derivatives were reported on their ethanolic (30% ethanol, *v/v*) and methanolic extracts (70% methanol, *v/v*) [63,68].

Focusing on their biological potential, hydroxycinnamic acids present antimicrobial, antioxidant and anti-inflammatory effects, being able to easily interact with detoxification and inflammation-related pathways, preventing the appearance or attenuating the development of many chronic diseases [52,54].

#### 3.2.2. Hydroxybenzoic Acids

The total content of hydroxybenzoic acids in *J. communis* L. leaves is relatively low [71]. As far as we know, protocatechuic acid was the only hydroxybenzoic acid reported in methanolic extracts (80% methanol, *v/v*) of *J. communis* var. *laxa* (c.a. 0.043 mg/g of dw) [60], while *ρ*-hydroxybenzoic, vanillic, gallic, and gentisic acids were found in methanolic extracts (80% methanol, *v/v*) of var. *saxatilis* at concentrations of 0.093, 0.060, 0.034, and 0.012 mg/g of dw, respectively [71]. Trace quantities of *ρ*-methylolphenol were also detected in methanolic extracts (100%, *v/v*) of *J. communis* (var. *communis*) [72]. Additionally, vestigial amounts of 2-hydroxybenzoic, 2,3-dihydroxybenzoic, ellagic and 4-*O*-methylgallic acids, and protocatechuic and gallic acid derivatives were reported in common *J. communis* L. berry ethanolic (30% ethanol, *v/v*) and methanolic (70% methanol, *v/v*) extracts [63,68].

As well as hydroxycinnamic acids, the hydroxybenzoic ones also display antimicrobial and antioxidant properties; however, they are less efficient given the lack of the CH=CH-COOH group and the double bond between carbons 7 and 8 [54].

#### 3.2.3. Flavan-3-ols

Among flavan-3-ols, catechin was one of the most predominant in *J. communis* L. leaves, with values ranging from 1.73 (methanolic extracts (80% methanol, *v/v*) of var. *laxa*) to 5.53 (methanolic extracts (80% methanol, *v/v*) of var. *saxatilis*) and 219.01 (var. *communis*) mg/g of dw [60,71,72]. Moreover, vestigial amounts of epicatechin, epigallocatechin, and catechin were also reported in methanolic extracts (80% methanol, *v/v*) of var. *saxatilis* and var. *communis* (<1.5 mg/g of dw) [71,72]. Flavan-3-ols were also detected on *J. communis* berry ethanolic (30% ethanol, *v/v*) and methanolic (70% methanol, *v/v*) extracts, namely (+)-gallocatechin 3-*O*-gallate, (−)-epicatechin, 3′-O-methyl(−)-epicatechin 7-*O*-glucuronide, (−)-epigallocatechin, 4′-*O*-methylepigallocatechin, 4″-*O*-methylepigallocatechin 3-*O*-gallate, cinnamtannin A2, and procyanidins dimer B1 and trimer C1, at trace amounts [63,68].

This phenolic’s subclass presents several health benefits, namely notable antimicrobial, antiparasitic, antiviral, antioxidant, anti-inflammatory, antiproliferative, cardioprotective, and neuroprotective properties [51,58].

#### 3.2.4. Flavonols

Within this subclass of compounds, the most abundant in *J. communis* L. leaves are quercetin aglycone and their derivatives, particularly quercetin 3-*O*-rutinoside [40,71,72]. In line with that, Fierascu et al. [40] reported quantities of 11.2 and 67.4 mg/g dw for quercetin and quercetin 3-*O*-rutinoside, respectively, in ethanolic extracts (50% ethanol, *v/v*) of common Romanian *J. communis* (var. n.s.). Methanolic extracts (80% methanol, *v/v*) of *J. communis* L. (var. *saxatilis*) also presented considerable amounts of quercetin 3-*O*-rutinoside (12.25 mg/g of dw) and small quantities of quercetin 3-*O*-glucoside (0.23 mg/g of dw), quercetin 3-*O*-rhamnoside (0.14 mg/g of dw), kaempferol 3-*O*-glucoside (0.021 mg/g of dw), and quercetin aglycone (<0.05 mg/g of dw) [71]. Regarding methanolic extracts (80% methanol, *v/v*) of var. *laxa*, they are also rich in quercetin 3-*O*-rutinoside (2.56 mg/g of dw) but present lower levels of kaempferol 3-*O*-rutinoside (0.22 mg/g of dw), kaempferol 3-*O*-glucoside (0.0097 mg/g dw), quercetin 3-*O*-glucoside (0.15 mg/g of dw), quercetin aglycone (0.11 mg/g dw), quercetin 3-*O*-rhamnoside (0.050 mg/g dw), isorhamnetin 3-*O-*rutinoside (0.079 mg/g dw), and isorhamnetin 3-*O*-glucoside (0.0037 mg/g dw) [60].

Concerning *J. communis* L. berry ethanolic (70% ethanol, *v/v*) and methanolic (70% methanol, *v/v*) extracts, they usually display many quercetin, kaempferol, myricetin, isorhamnetin, and patuletin derivatives in their composition [63,67]; particularly, methanolic extracts (100%, *v/v*) from var. *communis* and var. *saxatilis* present considerable amounts of quercetin hexoside (7.38 and 1.60 mg/g dw, respectively) [70]. Additionally, methanolic extracts (100%, *v/v*) of var. *communis* also exhibit trace amounts of quercetin 3-*O*-pentoside (1.01 mg/g dw) [70].

Given their chemical structure, flavonols are potent radical scavengers, and also show notable antimicrobial, anti-inflammatory, antiproliferative, and proapoptotic properties [54,68].

#### 3.2.5. Flavones

*J. communis* L. leaves also present some flavones in their constitution, highlighting the presence of apigenin on ethanolic extracts (50% ethanol, *v/v*) (13.2 mg/g dw) [40], and amentoflavone (0.39 mg/g dw) in methanolic extracts (80% methanol, *v/v*) of var. *saxatilis* [71]. Furthermore, this last variety also displays trace amounts of luteolin and naringenin (<0.1 mg/g dw) [71]. Methanolic extracts (80% methanol, *v/v*) of *J. communis* L. var. *laxa* exhibit vestigial amounts of apigenin (0.030 mg/g dw), luteolin 7-*O*-glucoside (0.011 mg/g dw), apigenin 7-*O*-glucoside (0.095 mg/g dw), and naringenin 7-*O*-glucoside (0.025 mg/g dw) [60]. The presence of apigenin 7-*O-*glucoside was also detected in methanolic extracts (100%, *v/v*) of var. *communis* [72]. On the other hand, apigenin, chrysoeriol, gossypetin derivatives, cirsilineol, isorhoifolin, luteolin, luteolin 3*-O*-galactoside, amentoflavone, and cupressoflavone were reported in *J. communis* L. berries [63,68,70]. For example, methanolic extracts (80% methanol, *v/v*) of berries from var. *communis* display considerable amounts of gossypetin hexoside and gossypetin hexoside-pentoside (amounts of 3.17 and 0.93 mg/g dw, respectively), hypolaetin 7-pentoside (ca., 7.99 mg/g dw), and isoscutellarein 7-*O*-pentoside (2.59 mg/g dw), while methanolic extracts (80% methanol, *v/v*) of var. *saxatilis* are richer in isoscutellarein 8-*O*-hexoside (2.44 mg/g dw), amentoflavone (0.95 mg/g dw), and methylbiflavone (0.85 mg/g dw) [70].

Although flavones are less effective in diminishing free radicals and reactive species levels due to the lack of the hydroxyl group at carbon 3 than other flavonoids, they display antimicrobial, antioxidant, and anticancer effects, as well as a notable ability to regulate lipid metabolism [54,64]. 

#### 3.2.6. Coumarins and Flavanones

Methanolic extracts (80% methanol, *v/v*) of *J. communis* L. leaf var. *saxatilis* present trace amounts of umbelliferone—which is a coumarin derivative—in its composition (c.a., 0.253 mg/g dw) [71], while methanolic extracts (80% methanol, *v/v*) of *J. communis* L. leaf var. *laxa* contain small quantities of taxifolin (flavanones) (0.0063 mg/g dw) [60].

Without surprises, both subclasses exhibit various biological activities, namely antioxidant, anti-inflammatory, anticancer, and anticoagulant properties [54,61]. Additionally, flavanones can act synergistically with flavones, inhibiting the development of estrogen-dependent colon cancers [25].

#### 3.2.7. Anthocyanins

Although *J. communis* L. leaves do not present anthocyanins, ethanolic extracts (30% ethanol, *v/v*) of their berries present some anthocyanins in their composition. Around 17 different anthocyanins were detected in berries, grouped as glycosides of cyanidin, delphinidin, peonidin, and pelargonidin [68].

Given their multiple hydroxyl groups, anthocyanins are potent radical scavengers, and also present notable anti-inflammatory abilities, being able to interact with related pathways, increase antioxidant defences, and diminish proinflammatory biomarkers, and in this way, prevent the occurrence of many oxidative-stress-related disorders [54,59].

### 3.3. Volatile Organic Compounds (VOC’s)

**Figure 4:**
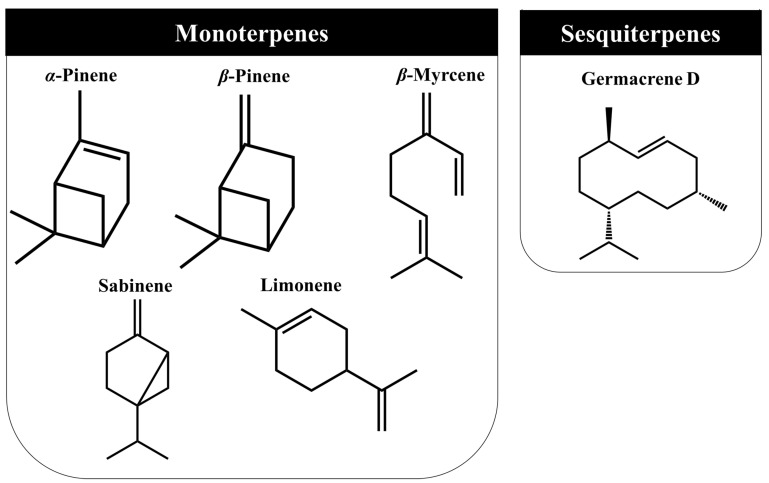
The main volatile organic compound found in *Juniperus communis* L. parts.

*J. communis* L. parts, namely their essential oils, present many volatile organic compounds (VOCs) in their composition, particularly the presence of monoterpene hydrocarbons, oxygenated monoterpenes, sesquiterpene hydrocarbons, and oxygenated sesquiterpenes [40,53]. As well as other phytochemicals, their levels also depend on genotype, origin, cultivation methods, meteorological conditions, and extraction techniques. Even so, among the species, monoterpenes such as *α*-pinene, *β*-pinene, and *β*-myrcene are the most commonly found, followed by some sesquiterpenes compounds, namely germacrene D (Figure 4) [73,74,75].

A total of 57 different VOCs were detected in leaf ethanolic extracts (50:50, *v/v*)*,* with pimaric acid being the predominant one (29.74% of total VOCs), followed by *α*-pinene (14.86% of total VOCs), *β*-myrcene (6.99% of total VOCs), bicyclosesquiphellandren (6.87% of total VOCs), and *β*-pinene (5.29% of total VOCs) [40]. On the other hand, extracts of var. *communis* exhibited higher percentages of limonene (26.12%), benzene (15.62%), *β*-myrcene (9.08%), and *β-*pinene (7.30%) [76]. Focusing on var. *alpina*, the predominant ones in their ethanolic extracts (50% ethanol, *v/v*) were *δ*-cadinene (12.80%), *α*-pinene (11.0%), germacrene D (9.30%), and borneol (8.60%) [77].

Regarding essential oils of their leaves, in the var. *communis*, *α*-pinene was also the most found (34.87% of total VOCs), followed by citronellyl acetate (14.26%), limonene (10.72%), and terpinolene (10.65%). Additionally, vestigial amounts (<6.21%) of *ρ*-cymene, elemene, cadinene, cyclohexane, cedrol, and caryophyllene were also reported [73]. *α*-Pinene was also the most abundant compound detected in different leaves of var. *communis* from different regions of the United States of America (USA) (66.6–75.2%) [78].

On the other hand, in leaves of var. *communis* from Serbia, sabinene was the main one reported (39.40%), followed by *α*-pinene (13.3%) and *β*-myrcene (4.70%) [79], whereas in var. *alpina* from France, limonene was clearly the most predominant (30.90%), followed by *α*-pinene (24.40%), *β*-phellandrene (12.60%), *β*-myrcene (3.60%), and *α*-phellandrene (3.60%) [80]. Similar percentages of *α*-pinene were reported on var. *saxatilis* (23.60%); additionally, this species was also shown to possess considerable percentages of *α*-cadinene (10.71%), sabinene (9.53%), germacrene D (7.25%), *α*-murolene (6.58%), and γ-cadinene (5.87%) [71,79].

Focusing on the berries of Portuguese *J. communis* var. *communis*, the main VOCs found in their essential oils were *α*-pinene (41.60%), *β*-pinene (27.60%), limonene (6.40%), *β*-myrcene (5.70%), and *trans*-pinocarveol (1.90%) [81]. A similar profile was found on Greek oil berries [82]. On the other hand, Iranian *J. communis* oils from the same species showed lower percentages of *α*-pinene (19.90%), and higher content of sabinene (36.8%) and limonene (10.60%) [83]. Additionally, essential oils from Iran also contained considerable percentages of germacrene D (8.10%) and terpinen-4-ol (3.60%) [83]. On the other hand, in Juniper oil berry var. *alpina* from Virginia (USA), *α*-pinene was the predominant one (20.00%), followed by δ-cadinene (10.40%), limonene and *β*-myrcene (8.70 and 8.50%, respectively) and borneol (8.00%) [82]. *a*-Pinene was also the main reported in var. *alpina* from Portugal (77.4%), followed by trace amounts of *β*-phellandrene (4.80%), *α*-terpinyl acetate (2.90%), and *β*-myrcene (2.60%) [84]. On the other hand, in oil berry var. *alpina* from France, limonene was the main found (49.30%), followed by *α*-pinene (22.10%) and *β*-myrcene (6.30%) [80]. Regarding oil berries, a high percentage of *α*-pinene was detected (51.40%) in common Romanian *J. communis*; additionally, myrcene, sabinene, limonene, and *β*-pinene were also found at percentages of 8.30, 5.80, 5.10, and 5.00%, respectively [32].

The combination of all of these results is evidence that local origin influences the phytochemical profile. Additionally, Gonny et al. [80] determined the VOC profile of *J. communis* woods and roots of var. *alpina*. For woods, *α*-terpinyl acetate (9.10%) and *α-*terpineol (8.4%) were the predominant ones, while for roots, a high percentage of cedrol (37.70%) and cinnamyl acetate (11.50%) were found.

VOCs have been gaining great interest owing to their remarkable antimicrobial, antioxidant, anti-inflammatory, and anticancer properties, being able to attenuate, or even mitigate, the development of cardiovascular disorders and neuropathologies, and also ameliorate the mental state of individuals [85].

## 4. Biological Potential of *Juniperus communis* Linnaeus

Since ancient times, *J. communis* parts have been largely used as antiseptics, contraceptives, and diuretics, and as a remedy to treat colds, chest complaints, rheumatism, headaches, dermatological and respiratory ailments, and kidney and urinary infections [38,39,85]. Given the aforementioned, it is not surprising that this plant is a focus of continuous studies to discover its full potential.

**Figure 5:**
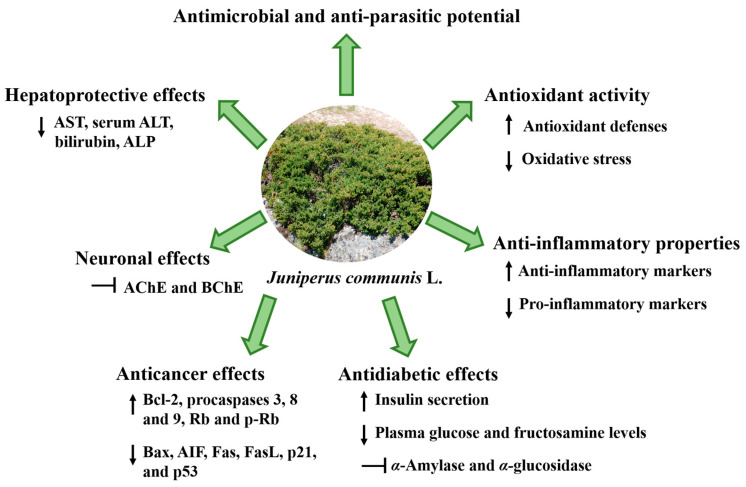
Main health-promoting properties attributed to *Juniperus communis* L. AST: aspartate aminotransferase; ALT: alanine aminotransferase; AChE: acetylcholinesterase; BChE): butyrylcholinesterase; Bax: Bcl-2-associated X protein; AIF: apoptosis-inducing factor; Fas: cell-surface death receptor; FasL: Fas ligand; Bcl-2: B-cell lymphoma 2; ↑: increase; ↓: reduction; T: inhibition.

To date, several reports have highlighted its antimicrobial, antifungal, antioxidant, anti-inflammatory, and antidiabetic potential, as well as its anticarcinogenic, hepatoprotective, neuronal, and renal effects, as described in Figure 5 and Table 2, Table 3 and Table 4 [34,38,40,66,86,87,88]. Next, a summary of the main studies already published concerning the health-promoting properties of this plant will be presented.

### 4.1. Antimicrobial, Antifungal, and Antiparasitic Potential

Antimicrobial and antiparasitic activity can be divided according to the nature of the employed extract, i.e., essential oils and phenolic-rich extracts, which in turn influence the different target activity, use, application, and range of microorganisms and parasitics inhibited [35] (Table 2). The use of essential oils is widespread in ethnobotanical phytotherapy, and for this reason, several works can be found [46]. Filipowicz et al. [89] analyzed the antibiotic capacity of different essential oils extracted from *J. communis* berries, each one with a specific composition. They concluded that the extract with a more balanced composition in its components (*α*-pinene, *β*-pinene, *p*-cymeno or limonene, among others) showed greater antibiotic effects against multiresistant hospital isolates belonging to the species *Staphyllococcus aureus*, *Serratia marcescens*, *Enterobacter cloace*, *Klebsiella pneumoniae*, *Pseudomonas aeruginosa*, *Acinetobacter baumanii*, and *Listeria monocytogenes*, as well as in *Candida albicans.* The authors also verified that there effectively existed a synergistic effect between all components of the oil. 

On the other hand, essential oils from *J. communis* needles (var. *alpina*) are shown to have notable effects in inhibiting the growth of numerous dermatophyte fungi (*Epidermophyton floccosum*, *Microsporum canis*, *M. gypseum*, *Trichophyton mentagrophytes*, *T. mentagrophytes* var. *interdigitale*, *T. rubrum*, and *T. verrucosum*), with active concentrations ranging between 0.32 to 2.5 µL/mL (*vs.* inhibition values of 16 and 128 µg/µL for the antifungal fluconazole) [90]. However, essential oils of leaves and fruits of *J. communis* (var. *communis*) from Sardinia exhibited weak antibiotic activity against *C. albicans*, *S. aureus*, and *P. aeruginosa* (Minimum Inhibitory Concentrations (MICs) higher than 1 mg/mL) [91]. Even so, it was observed that the use of pure solutions of juniper essential oils showed lower activity than the solutions diluted in at least 50% ethanol; this evidence is probably due to an improvement in the solubility of essential oils, which in turn increase its effectiveness [36].

These results agree with those obtained in Slovenia using distillates obtained through medium-scale industrial processes. Here, the essential oils of *J. communis* were able to inhibit the development of *S. aureus* and *C. albicans*, both of type strains and clinical isolates, showing in the latter case inhibition halos of 7.00 ± 0.01 mm and 21.33 ± 0.88 mm, respectively [92].

Furthermore, the use of essential oils obtained from *J. communis* biomass, without differentiating each of its parts, showed remarkable inhibitory activity against *Escherichia (E.*) *coli*, at concentrations between 1.25 and 2.5 mg/mL. As expected, there were observed variations regarding the obtained data due to the different collection sites and consequent different edaphoclimatic conditions, which in turn influenced the essential oil extracts’ composition [75]. On the other hand, no notable inhibitory activities were observed against other Gram-negative bacteria, such as *Proteus mirabilis*, *K. pneumoniae*, *P. aeruginosa*, and *Morganella morganii*; however, a slight activity against *L. monocytogenes* and methicillin-resistant *S. aureus* was observed [75]. Similar results were obtained comparing the activity of commercial *J. communis* berry essential oils and hydrodistilled berry extracts from wild Portuguese plants, observing a considerable variation in the MIC, minimum bactericidal concentration (MBC), and minimum fungicidal concentration (MFC). The obtained data were expressed in % *v/v* between each of the extracts, and even without showing susceptibility to the highest concentrations tested (2.5% *v/v*), greater susceptibility was seen to Gram-positive species (*B. cereus*, *B. subtilis* and *S. aureus*) than Gram-negative species [81]. The study of the antibiotic capacity of the essential oils of *J. communis* leaves against 16 species of bacteria and 14 species of fungi, some of them dermatophytes fungus, showed similar results to those already observed for bacteria, where Gram-positive ones had a greater susceptibility than Gram-negative ones, with MIC and MBC varying between 8 and 70% *v*/*v*. The MIC results observed for fungi ranged between 0.39 and 10% *v*/*v*, while MBC values were between 0.78 and 12.5% *v*/*v* [26]. The essential oils from *J. communis* fruits (var. *alpina*) showed an outstanding activity against different types of pathogenic fungi, with MIC values ranging between 1.25 and 20 µL/mL; the highest susceptibilities were found against dermatophyte fungi, such as *M. canis*, *T. rubrum*, or *E. floccosum* [84]. These results are similar to those obtained with essential oils of *J. communis*, with MIC values of 11.11 mg/mL and 13.98 mg/mL for different *Aspergillus* species and between 11.11 mg/mL and 12.50 mg/mL for species of the genus *Penicillium*, which are also 2 times higher than those obtained for the commercial antifungal ketoconazole [93].

The analysis of the volatile fraction of *J. communis* essential oils presented similar results to those observed in essential oils and phenolic-rich extracts, with an intense activity against Gram-positive bacteria such as *S. aureus,* but not against Gram-negative ones such as *E. coli*. In fact, a concentration about 3 times lower was necessary to inhibit *S. aureus* growth (4.75 µg/mL) compared to *E. coli* (16.8 µg/mL). The comparison between the volatile composition and other constituents of the *Juniperus* genus showed that there may exist a relationship between the quantity of sesquiterpene hydrocarbons and aromatic oxygenated hydrocarbons, mostly found in the extract of *J. communis,* and their effectiveness against Gram-positive bacteria [94]. Even so, the use of the volatile fraction extracted from berries of var. *alpina*, mainly composed of *α*-pinene and *δ*-3-carene, showed a lower MIC than the complete extract, at concentrations ranging from 0.16 to 1.25 µL/mL [84]. In this regard, it has been described that the antibiotic capacity of *α*-pinene depends on the enantiomeric properties of this compound, cultivation origin, and part of the plant (leaves or cones) [95].

**Table 2:** Antimicrobial and antiparasitic activity of different *Juniperus communis* extracts.

Part of the Plant	Origin	Subspecies/Variety	Method	Inhibited Species	References
Antimicrobial activity					
Essential oils					
Berries	Poland	n.s.	Disc diffusion	*Staphyllococcus aureus, Serratia marcenscens, Enterobacter cloace, Klebsiella pneumoniae, Pseudomonas aeruginosa, Acinetobacter baumanii, Listeria monocytogenes,* and *Candida albicans*	[89]
Needles	Portugal	var. *alpina*	MIC and MLC	*Epidermophyton floccosum, Microsporum canis, M. gypseum, Trichophyton mentagrophytes, T. mentagrophytes var. interdigitale, T. rubrum,* and *T. verrucosum*	[90]
Needles and berries	Italy	var. *communis*	MIC	*C. albicans, S. aureus,* and *P. aeuroginosa*	[91]
Plant material (leaves and stems)	Iran	n.s.	Disc diffusion	*S. aureus, P. aeruginosa,* and *E. coli*	[36]
Berries	Slovenia	n.s.	Biofims assay	*Campylobacter jejuni, L. monocytogenes*	[96]
Plant material (undifferentiated)	Slovenia	n.s.	Disc diffusion	*S. aureus* and *C. albicans*	[92]
Berries	Spain	n.s.	MIC	*E. coli, Proteus mirabilis, K. pneumoniae, P. aeruginosa and Morganella morganii,* MRSA, and *L. monocytogenes*	[75]
Berries	Portugal	n.s.	MIC and MLC	*B. cereus, B. subtilis, E. aerogenes, E. faecalis, E. coli, K. pneumoniae, Proteus mirabilis, P. aeruginosa, Salmonella typhimurium, S. aureus,* and *C. albicans*	[81]
Leaves	Croatia	n.s.	Disc diffusion, MIC, and MLC	16 species of bacteria and 14 species of fungus	[26]
Berries	Serbia	n.s.	Disc diffusion, MIC, MLC, and in vivo adhesion assay	*S. aureus, MRSA, E. faecalis, L. monocytogenes, E. coli, S. flexneri, S. enteritidis, P. aeruginosa, Aspergillus fumigatus, A. versicolor, A. ochraceus, A. niger, Trichoderma viride, Penicillium funiculosum, P. ochrochloron,* and *P. verrucosum* var. *cyclopium*	[93]
Plant material (leaves and branches)	Egypt	n.s.	MIC	*S. aureus, E. coli,* and *C. albicans*	[94]
Plant material	Croatia	n.s.	MIC and biofilm assay	*Mycobacterium avium, M. intracellulare,* and *M. gordonae*	[97,98]
Phenolic-rich extracts					
Berries	Slovenia	n.s.	Biofilms assay	*C. jejuni, L. monocytogenes*	[96]
Plant material	Italy	n.s.	Disc diffusion and MIC	*Actinomyces viscosus, Lactobacillus casei, Streptococcus mutans, S. sobrinus,* and general oral microbiota	[99]
Berries	Turkey	n.s.	Disc diffusion and MIC	*S. epidermidis, S. aureus, B. subtilis, P. aeruginosa, E. coli,* and *C. albicans*	[100]
Leaves	Turkey	var. *communis* and var. *saxatilis*	MIC	*S. aureus*	[101]
Leaves	Poland	n.s.	Disc diffusion	*K. pneumoniae, S. enteritidis,* *P. aeruginosa, A. baumannii, E. faecium, S. aureus, L. fermentum, Clostridium butyricum, L. monocytogenes, B. coagulans, C. utilis, Aspergillus* spp., and *Fusarium* spp.	[102]
Stem (branches)	Italy	var. *communis* and var. *saxatilis*	Biofilm formation	*S. aureus*	[103]
Berries	Turkey	var. *communis* and var. *saxatilis*	MIC and MLC	*S. aureus, S. epidermidis, E. hirae, B. subtilis, E. coli, P. mirabilis, P. aeruginosa, C. albicans,* and *C. parapsilosis*	[70]
Leaves	India	n.s.	MIC	*E. coli, S. aureus,* and *K. pneumoniae*	[104]
Antiparasitic activity					
Essential oils					
Stems and leaves	France	n.s.	Radioactive micromethod	Two different strains of *Plasmodium falciparum*, which were chloroquine-resistant (FcBl) and chloroquine-sensitive (Nigerian) strains	[105]

n.s.: not specified; MIC: Minimal inhibitory concentration; MLC: Minimal lethal concentration.

In another way, the use of subinhibitory concentrations (1 mg/mL) of juniper-fruit essential oils has been shown to possess a severe effect as an antiadhesion agent in *Campylobacter jelunni*, preventing the formation of biofilms by up to 100% to concerning the control on plastic surfaces [96]. The use of essential oils of *J. communis* from the whole plant (leaves and branches) has been shown to have a temperature-dependent growth-inhibitory effect (MIC) for bacteria of the genus *Mycobacterium*, at concentrations between 0.8 and 3.2 mg/mL. Although the use of subinhibitory concentrations allows limiting the formation of biofilms by up to 49% after 3 days [98], its combination with essential oils extracted from *Helichrysum italicum* has been shown to have synergistic activity, allowing the concentrations used to be reduced 3-fold to achieve similar effects [97]. It has also been observed that the use of essential oils from *J. communis* berries is capable of reducing the adhesion of *L. monocytogenes* cells to HT-29 and HCT116 colon-cancer cells by 62%, thus reducing the capacity of this foodborne pathogen to cause intracellular infections by establishing favorable and competent unions with the host cells [93].

Comparing a total of 72 different plants used in European ethnobotany, the hexane extract of *J. communis* leaves was one of the most effective in inhibiting pathogenic microorganisms present in the oral microbiota, with higher inhibition halos in diffusion-inhibition tests and a greater range of inhibited microorganisms [99]. On the other hand, the ethanolic extract rich in phenolic compounds from *J. communis* seeds showed a discrete antibiotic activity, with inhibition halos ranging between 7 and 12 mm at solutions of 100 mg/mL dw, presenting a higher sensitivity against Gram-positive (*S. epidermidis, S. aureus, B. subtilis*) than Gram-negative (*P. aeruginosa, E. coli*) and fungal (*C. albicans*) strains. These data were supported by the obtaining MIC, yielding a result of 3.125 µg/mL for the three Gram-positive species when compared to values that varied between 12.5 µg/mL and 50 µg/mL regarding the other studied species [100]. The comparison between methanolic and aqueous phenolic-rich extracts from leaves of two different *J. communis* varieties (var. *communis* and var. *saxatilis*) revealed that the methanolic extract presents a higher activity, requiring lower concentrations to inhibit the growth of *S. aureus* (78.12 µg/mL and 39.06 µg/mL, respectively) when compared to the aqueous extracts, which required much higher concentrations (1250 µg/mL and 312.5 µg/mL respectively). Additionally, it was observed that the var. *saxatilis* was more active than var. *communis* [101]. Additionally, berries’ phenolic extracts from var. *communis* and var. *saxatilis* showed that although the var. *communis* had a higher concentration of phenolic compounds, the MIC and MBL were lower for the var. *saxatilis* against *S. aureus* (156.25 µg/mL), *S epidermidis* (1250 µg/mL), *Enterococcus hirae* (156.25 µg/mL), and *B. subtilis* (156.25 µg/mL), compared with var. *communis* (*S. aureus* (156.25 µg/mL), *S epidermidis* (1250 µg/mL), *En. hirae* (625 µg/mL), and *B. subtilis* (321.5 µg/mL) [70]. On the other hand, the use of aqueous extract rich in phenolics against different bacteria and fungi showed around 50% more activity in disk-inhibition tests against Gram-positive bacteria (*Lactobacillus fermentum*—17 mm, *S. aureus*—15 mm and *L. monocytogenes*—15 mm) than against Gram-negative (*P. aeruginosa*—10 mm and *Acinetobacter baumannii*—11 mm). In the case of fungi, very discrete inhibition activities were observed (*C. utilis*—3 mm, *Aspergillus* sp.—6 mm and *Fusarium* sp.—2 mm) [102].

Another possibility is the combination of these extracts rich in phenolic compounds with routinely used commercial antibiotics such as tetracycline, chloramphenicol, and erythromycin. It was already reported that their combination with alcoholic extracts rich in phenolic compounds from *J. communis* leaves can effectively improve their efficiency against *E. coli*, *S aureus*, and *K. pneumoniae* by between 2 and 525 times, allowing a considerable reduction in the MIC of the antibiotic [104].

The use of subinhibitory concentrations (1 mg/mL) of ethanolic extracts and essential oils of juniper berries can also have a severe effect as an antiadhesion agent in *Campylobacter jeunni*, preventing the formation of biofilms by up to 95% on plastic surfaces [96]. In this way, the use of methanolic and aqueous extracts of *J. communis* var. *communis* and var. *saxatilis* branches can inhibit the growth of *S. aureus*. The methanolic extract showed reductions in initial adhesion after 3 h (22% in var. *saxatilis* and 44% in var. *communis*) and in the formation of biofilms 24 h after inoculation (66% in var. *saxatilis* and 68% in var. *communis*). Meanwhile, the aqueous extract proved to be more active in controlling biofilm formation, limiting the initial adhesion of bacterial cells to surfaces (25% in var. *communis* and 50% in var. *saxatilis*) and the extent of the biofilm formed (81% in var. *communis* and 84% in var. *saxatilis*) [103].

Concerning antiparasitic potential, essential oils extracted from leaves and stems of *J. communis* showed potential to inhibit the growth of two malarial strains different to *Plasmodium falciparum*, which were chloroquine-resistant (FcBl) and chloroquine-sensitive (Nigerian) strains, exhibiting in both cases an IC_50_ value of 1 mg/mL after 24 and 72 h of exposure. No cumulative effects were found over time [105]. This activity is mainly due to the presence of *α*-pinene, which is one of the main essential oils extracted from this plant. In fact, this terpene has already been shown to possess notable antimalarial activity (IC_50_ value of 1.2 µM) [106].

### 4.2. Antioxidant Activity

The antioxidant power of *J. communis* was established using different in vitro antioxidant assays, such as 2,2-diphenyl-1-picrylhydrazyl (DPPH), 2,2’-azino-bis (3-ethylbenzothiazoline-6-sulfonic acid) (ABTS), superoxide anion and hydroxyl radical scavenging assays, and metal-chelating potential, as well as by in vivo assays (Table 3).

#### 4.2.1. In Vitro Studies

Focusing on in vitro tests, and concerning the DPPPH^●^, the ethanolic extract of berries showed a half-maximal inhibitory concentration (IC_50_) of 1.42 µg/mL [40]. Additionally, its ethanol, ethyl acetate, and chloroform extracts revealed IC_50_ values of 28.55, 106.44, and 257.66 µg/mL, respectively [50]. Berries’ methanolic extracts and their essential oils also exhibited the capacity to scavenge DPPH^●^ (IC_50_ varying between 6.86 and 13.66 µg/L, and from 1.27 to 4.25 µg/L, respectively), ferric species (reducing power ranging 6.90 and 10.70 mM FeSO_4_·7H_2_O for the methanolic extract and between 0.47 and 1.11 mM FeSO_4_·7H_2_O for the essential oil), and *β*-carotene species (24.36–30.63% for the methanolic extract and 1.19–2.39% concerning the essential oil) [63]. Berries’ ethanolic extracts also reveal the capacity to scavenge hydroxyl radicals, showing inhibitory values ranging from 65.59 to 88.12% for crushed and between 15.52 and 32.85% for noncrushed berries [67]. Ethanolic extracts of *J. communis* berries also revealed the ability to scavenge peroxyl radicals (3876 µM Trolox equivalents at a concentration of 1 mg/mL) [69] and reduce power potential (12.82 ascorbic acid equivalent/mL) [70], while its essential oil can scavenge ABTS^•+^ species (IC_50_ value of 10.96 µg/mL) and superoxide anions (IC_50_ of 0.822 µg/mL), and remove hydroxyl radicals before they can degrade deoxyribose (IC_50_ = 0.0066 µg/mL) [32]. Ethanolic, aqueous, and ethyl acetate fractions of *J. communis* leaves can also reduce DPPH^●^ (IC_50_ values of 213, 347, and 177 µg/mL, respectively) [65], while its methanolic extracts showed an IC_50_ value of 258 µg/mL [60]. On the other hand, their ethanolic and ethyl acetate fractions can chelate ferric species (IC_50_ scores of 654 and 261 µg/mL, respectively) [65]. Acetate extracts revealed the potential to chelate metals, displaying an inhibitory effect of 6.05% at 1 mg/mL [107]. Ethanolic leaf extracts also showed the capacity to capture superoxide anions at concentrations of 0.5, 1 and 2 mg/mL, revealing inhibitory percentages of 20.26, 25.00 and 25.38%, respectively [61]. In addition, their distilled extracts and essential oils showed lipid-peroxidation inhibitory potential (IC_50_ values of 540 and 2440 µg/mL, respectively), while distilled extracts showed a ferric reduction activity of 78.77 mg of ascorbic acid equivalents per g of dw [71]. Essential oils of its leaves revealed an IC_50_ score of 660 µg/mL regarding the capture of DPPH^●^ [79]. In the ABTS^●+^ assay; hydroethanolic extracts of its leaves and fruits revealed inhibitory percentages of 99.5 and 42.5%, respectively, at 3000 µg/mL [66].

On the other hand, *J. communis* shoots showed potential to reduce reactive oxygen species and increase the activity of intracellular antioxidant-enzyme superoxide dismutase and catalase [56]. Furthermore, its acetone, ethyl acetate, and ethanol extracts showed inhibitory percentages of 6.05, 22.59, and 12.31%, respectively, at 1 mg/mL regarding metal-chelating potential [107]. In addition, essential oils of its twigs can inhibit peroxy-radical-induced oxidation, exhibiting values of around 120 µmol Trolox/gram of essential oil [53]. Ethanolic extracts of hops also displayed ferric-ion-reducing antioxidant power (4.17 mg of ascorbic acid equivalents per g), and the capacity to capture DPPH^●^ and ABTS^●+^ species (9.26 and 49.54 mg of ascorbic acid equivalents per g, respectively) [68].

#### 4.2.2. In Vivo Studies

The administration of methanolic extracts of this plant (200 mg/kg) for 21 days on chlorpromazine-induced Parkinson’s disease in rats also showed increments in reduced glutathione and decreased levels of TBARS as compared to the untreated group [19]. Furthermore, the inhalation of its oil for 60 min daily for 21 days revealed higher levels of superoxide dismutase and catalase enzymes, and glutathione peroxidase activity on rats’ hippocampus subjected to amyloid *β* (1–42)-induced oxidative stress [85].

The remarkable antioxidant abilities showed by *J. communis* L. species are intimately linked to their phenolic and terpenoid content, in particular the presence of quercetin aglycone and their derivatives [32,67,69,71,79]. This flavan-3-ol possesses several hydroxyl groups in its constitution, which makes it a potent radical scavenger. As evidence, positive correlations (*r* > 0.80; *p* < 0.05) were already reported concerning their levels and capacity to neutralize DPPH^●^ [108] and to inhibit lipid peroxidation in human erythrocytes [109,110]. Regarding terpenes’ antioxidant activity, Burits and colleagues [111] already reported that *α*-pinene, *ρ*-cymene, limonene, and linalool possess notable capacities to block lipid peroxidation (IC_50_ values of 0.51, 0.69 and 0.67 µL/mL, respectively) and to avoid deoxyribose degradation (IC_50_ scores of 0.78, 0.91 and 0.28 µL/mL, respectively). Similar potential was also reported by Emamia and collaborators [112] concerning *β*-pinene, cedrol, and sabinene antioxidant potential. Moreover, this property also depends on the extraction solvents applied, usually being higher when water–alcohol mixtures are used, given their great affinity for both lipophilic and hydrophilic bioactive molecules [50,59].

**Table 3:** In vitro and in vivo antioxidant effects of *Juniperus communis* extracts.

Part of the Plant	Origin	Extract	Subspecies/ Variety	Experimental Model	Effect	References
In vitro assay						
Berries	Romania	Ethanolic (50% ethanol, *v/v*)	n.s.	Capacity to scavenge DPPH^●^	IC_50_ value of 1.42 µg/mL	[32,40,50,61,67]
	Serbia	Ethanolic			IC_50_ value of 28.55 µg/mL	
		Ethyl acetate			IC_50_ value of 106.44 µg/mL	
		Chloroform			IC_50_ value of 257.66 µg/mL	
	Poland	Methanolic (70%, methanol *v/v*)			IC_50_ values from 6.86 to 13.66 µg/L	
		Essential oils			IC_50_ varying from 1.27 to 4.25 µg/L	
	Turkey	Methanolic	var. *saxatilis*		IC_50_ value of 1.84 mg/mL	
			var. *communis*		IC_50_ value of 0.63 mg/mL	
		Ethanolic (80% ethanol, *v/v)*	var. *alpina*		Inhibitory percentages of 33.25, 34.27, and 36.26% at 0.5, 1, and 2 mg/mL, respectively	
		Aqueous			Inhibitory percentages of 48.40, 63.29, and 82.03% at 0.5, 1, and 2 mg/mL, respectively	
	Poland	Methanolic (70% methanol, *v/v*)	n.s.	Reducing power potential	Values ranging 6.90 and 10.70 mM FeSO_4_ × 7H_2_O	[61,63,67,70]
		Essential oils			Values ranging from 0.47 and 1.11 mM FeSO_4_ × 7H_2_O	
	Turkey	Methanolic	var. *communis*		IC_50_ value of 12.82 mg/mL	
					12.82 ascorbic acid equivalent/mL	
			var. *saxatilis*		IC_50_ value of 64.14 mg/mL	
					64.14 ascorbic acid equivalent//mL	
		Ethanolic (80% ethanol, *v/v)*	var. *alpina*		Inhibitory percentages of 0.083, 0.095, and 0.203% at 0.5, 1, and 2 mg/mL, respectively	
		Aqueous			Inhibitory percentages of 0.424, 0.689, and 1.371% at 0.5, 1, and 2 mg/mL, respectively	
	Poland	Methanolic (70% methanol, *v/v*)	n.s.	*β-*carotene bleaching test	*β-*carotene inhibitory potential varying from 24.36 to 30.63%	[63]
		Essential oils	n.s.		*β-*carotene inhibitory potential varying from 1.19 to 2.39%	
	Turkey	Methanolic	var. *saxatilis*	Protect liposomes from lipid peroxidation	IC_50_ value of 120.07 µg/mL	[70]
			var. *communis*		IC_50_ value of 4.44 µg/mL	
	Turkey	Methanolic	var. *saxatilis*	Ferrous ion (Fe^2+^)-chelating activity	Chelating ability around 30% at 2 mg/mL	[32,61,69]
			var. *communis*		Chelating ability around 15% at 2 mg/mL	
		Ethanolic (80% ethanol, *v/v)*	var. *alpina*		Inhibitory percentages of 4.88, 14.86, and 32.82% at 0.5, 1, and 2 mg/mL, respectively	
		Aqueous			Inhibitory percentage of 0.83% at 2 mg/mL	
	Canada	Ethanolic (80% ethanol, *v/v*)	var. *communis*	Capacity to scavenge peroxyl radicals	3876 µM Trolox equivalents at 1 mg/mL	[32,66]
	n.s.	Essential oil	n.s.	Capacity to scavenge ABTS^•+^ species	IC_50_ value of 10.96 µg/mL	
	Turkey	Ethanolic (80% ethanol, *v/v*)	var. *saxatilis*	Capacity to scavenge ABTS^•+^ species	Inhibitory percentages of 42.5%, respectively at 3 mg/mL	
	n.s.	Essential oil	n.s.	Capacity to scavenge hydroxyl radicals	IC_50_ value of 0.0066 µg/mL	[32]
	n.s.	Essential oil	n.s.	Capacity to scavenge superoxide anions	IC_50_ of 0.822 µg/mL	[32,61]
	Turkey	Ethanolic (80% ethanol, *v/v)*	var. *alpina*		Inhibitory percentages of 20.07, 21.97, and 17.80% at 0.5, 1, and 2 mg/mL, respectively	
		Aqueous	var. *alpina*		Inhibitory percentages of 5.49, 10.61, and 11.17% at 0.5, 1 and 2 mg/mL, respectively	
Crushed berries	Slovakia	Ethanolic (70% ethanol, *v/v*)	n.s.	Capacity to scavenge hydroxyl radicals	Inhibitory values varying from 65.59 to 88.12% (recalculated by dry matter (DM), from 3.06 to 5.75%/g DM)	[67]
Noncrushed berries	Slovakia	Ethanolic (70% ethanol, *v/v*)	n.s.		Inhibitory values varying from 15.52 and 32.85% (recalculated by dry matter (DM), from 1.20 to 20.05%/g DM) for	
Unripe berries	Turkey	Ethanolic (80% ethanol, *v/v)*	var. *alpina*	Capacity to scavenge superoxide anions	Inhibitory percentages of 14.58, 10.99, and 18.37% at 0.5, 1, and 2 mg/mL, respectively	[61]
				Capacity to scavenge DPPH^●^	Inhibitory percentages of 46.21, 57.32, and 73.75% at 0.5, 1, and 2 mg/mL, respectively	[32,61]
				Capacity to chelate metals	Inhibitory percentages of 6.32, 5.04, and 16.59% at 0.5, 1, and 2 mg/mL, respectively	[61]
				Ferric-reducing antioxidant power	Inhibitory percentages of 0.288, 0.504, and 0.855% at 0.5, 1, and 2 mg/mL, respectively	
Leaves	India	Ethanolic (70% ethanol, *v/v*)	n.s.	Capacity to scavenge DPPH^●^	IC_50_ value of 213 µg/mL	[60,61,65,79,107]
		Aqueous	var. *communis*		IC_50_ value of 347 µg/mL	
		Ethyl acetate			IC_50_ value of 177 µg/mL	
	Turkey	Ethanolic (80% ethanol, *v/v)*	var. *alpina*		Inhibitory percentages of 66.62, 83.06, and 91.40% at 0.5, 1, and 2 mg/mL, respectively	
		Aqueous			Inhibitory percentages of 34.92, 35.56, and 37.29% at 0.5, 1, and 2 mg/mL, respectively	
	Bulgaria	Methanolic (80% methanol, *v/v*)	var. *Oblonga Pendula*		IC_50_ value of 258 µg/mL	
	Serbia	Essential oil	var. *communis*		IC_50_ value of 660 µg/mL	
	Serbia	Essential oil	var. *saxatilis*	Potential to chelate metals	IC_50_ value of 320 µg/mL	[61,107]
	India	Ethyl acetate	var. *communis*		IC_50_ value of 261 µg/ mL	
	Turkey	Acetate	n.s.		Inhibitory effect of 6.05% at 1 mg/mL	
		Aqueous	var. *alpina*		Inhibitory percentages of 9.06, 12.39, and 38.40% at 0.5, 1, and 2 mg/mL, respectively	
	Turkey	Ethanolic (80% ethanol, *v/v)*	var. *alpina*	Capacity to scavenge superoxide anions	Inhibitory percentages of 20.26, 25.00, and 25.38% at 0.5, 1, and 2 mg/mL, respectively	[61,71]
	Turkey	Ethanolic (80%, ethanol *v/v)*	var. *alpina*	Ferric-reducing antioxidant power	Inhibitory percentages of 0.681, 1.278, and 1.971% at 0.5, 1, and 2 mg/mL, respectively	
		Aqueous			Inhibitory percentages of 0.121, 0.120, and 0.154% at 0.5, 1, and 2 mg/mL, respectively	
	Serbia	Distilled extracts	var. *saxatilis*		Reduction capacity of 78.77 mg of ascorbic acid equivalents per g of dry matter	
	Serbia	Distilled extracts	var. *saxatilis*	Lipid-peroxidation inhibitory potential	IC_50_ value of 540 µg/mL	[71]
		Essential oils			IC_50_ value of 2440 µg/mL	
	Turkey	Ethanolic (80% ethanol, *v/v*)	var. *saxatilis*	Capacity to scavenge ABTS^•+^ species	Inhibitory percentage of 99.5 at 3 mg/mL	[66]
Shoots	Poland	Crude extract	n.s.	Antioxidant-enzyme activity and reactive oxygen species in vitro assays	↑↑ the activity of intracellular antioxidant enzymes superoxide dismutase and catalase ↓↓ reactive oxygen species	[56]
	Turkey	Acetone	n.s.	Capacity to chelate metals	Inhibitory percentage of 6.05% at 1 mg/mL	[107]
		Ethyl acetate			Inhibitory percentage of 22.59 at 1 mg/mL	
		Ethanolic (75% ethanol, *v/v*)			Inhibitory percentage of 12.31% at 1 mg/mL	
Twigs	Spain	Essential oil	n.s.	Peroxy-radical-induced oxidation inhibition	120 µmol Trolox/gram of essential oil	[53]
Hops	Australia	Ethanolic (30% ethanol, *v/v*)	n.s.	Ferric ion-reducing antioxidant power	4.17 mg of ascorbic acid equivalents per g	[68]
				Capacity to scavenge DPPH^●^	9.26 mg of ascorbic acid equivalents per g	
				Capacity to scavenge ABTS^•+^ species	49.54 mg of ascorbic acid equivalents per g	
Plant material (twigs, leaves, and berries)	Spain	Essential oil	n.s.	Reducing power assay	IC_50_ values from 135 to 970 µg/mL	[75]
	Spain	Essential oil	n.s.	Inhibition of oxidation process	IC_50_ values from 324.76 to 1563.29 µg/mL	
In vivo assay						
Leaves	India	Methanolic	n.s.	Effects on Wistar rats with induced Parkinson’s disease by chlorpromazine for 21 days at a dose of 200 mg/kg	↑↑ in reduced glutathione ↓↓ levels of TBARS	[19]
	Romania	Essential oil	n.s.	Effects of juniper volatile oil (1% and 3%) daily inhalation on Amyloid Beta (1–42)-induced oxidative stress in Wistar rats	↑↑ superoxide dismutase and catalase enzymes, and glutathione peroxidase activity	[85]

n.s.: not specified; IC50: half-maximal inhibitory concentration; TBARS: thiobarbituric acid-reactive substances, DPPH^●^: 2,2-diphenyl-1-picrylhydrazyl radical; ABTS^•^+: 2,2′-azino-bis (3-ethylbenzothiazoline-6-sulfonic acid; ↑↑: increase; ↓↓: reduction.

### 4.3. Anti-Inflammatory and Antinociceptive Properties

The anti-inflammatory effects of this plant have already been evaluated by in vitro and in vivo studies.

#### 4.3.1. In Vitro Studies

By in vitro studies, it was already mentioned that aqueous extracts of *J. communis* can inhibit prostaglandins by 55% at 200 µg/mL and platelet-activating factor-induced exocytosis by 78% at 250 µg/mL [25]. Moreover, Schneider et al. [88] also revealed that methylene chloride extracts of its woods and berries, and berry ethyl acetate extract at 100 µg/mL can effectively reduce the production of 12[S]-hydroxy-5,8,10,14-eicosatetraenoic acid by 54.0, 66.2, and 76.2%, respectively. Essential oils of its plant material (twigs, leaves, and fruits) from two different Spanish regions also showed potential to inhibit the lipopolysaccharide-induced nitric oxide production on RAW 264.7 murine macrophage cells (IC_50_ values of 84.80 and 23.98 µg/mL for the regions of Almazán and Barriomartín, respectively) [75]. From the methylene chloride extract of the wood were extracted cryptojaponol and *β*-sitosterol, which in turn showed inhibitory activities of 55.4 and 25.0% regarding 12[S]-hydroxy-5,8,10,14-eicosatetraenoic acid production, respectively, at concentrations of 100 µg/mL.

#### 4.3.2. In Vivo Studies

Focusing on in vivo studies, Mascolo and collaborators [113] screened the anti-inflammatory potential of hydroethanolic extracts of 27 plants from different families largely used in Italian folk medicine and reported that *J. communis* was one of the most effective in reducing the rats’ swelling-foot edema induced by carrageenin. Indeed, the obtained data revealed that at doses of 100 and 200 mg/kg and after 7 days of treatment, a reduction was verified regarding carrageenin-foot edema by 60% and 79%, respectively, against 45% shown by positive-control indomethacin (5 mg/kg). Similar results were reported by Akkol et al. [114]. Additionally, Akkol and coworkers [114] also verified that these extracts also revealed anti-inflammatory potential in PGE-2-induced hind-paw edema in a pattern similar as the carrageenin-edema model. More recently, the anti-inflammatory potential of *J. communis* was assessed using two different inflammation experimental models (dextran and kaolin), and it was verified through plethysmometry that the treatment with hydroethanolic microemulsions of *J. communis* berries can effectively reduce paw edema in the dextran-induced inflammation model, mainly due to its antihistaminic and antiserotonin activities. On the other hand, in the kaolin-induced inflammation model, the administration of this microemulsion showed potential to significantly downregulate the expression of proinflammatory interleukins (IL)-1*β* and IL-6, and tumor necrosis factor alfa, owing to its content in phenolics [40].

Beyond what was reported, Banerjee and colleagues [29] revealed that methanolic extracts of *J. communis* leaves possess analgesic effects. The authors conducted an in vivo study involving different nociceptive assays (acetic acid-induced writhing, formalin, and tail-flick tests) in rodents and verified that the extract administration of 100 mg/kg and 200 mg/kg can significantly inhibit, in a dose-dependent manner, the writhing response and the late phase related with the formalin test as compared to aspirin. Furthermore, it was also verified that this plant can act centrally, since the extract and pethidine effects were blocked by naloxone in the tail-flick test.

Nowadays, it is well-accepted that anti-inflammatory and antianalgesic activities of *J. communis* parts are intimately associated with the presence of phenolic compounds and terpenes, namely *α*-pinene, 1-octanol, amentoflavone, and linalool, whose capacity to inhibit inflammatory cytokine and prostaglandin expression was already known [40,115,116,117]. Particularly, hydroxybenzoic acids (25 µM), caffeic acid (10 µM), *ρ*-coumaric acid (50 µM), and quercetin (100 µM) already revealed the ability to interfere with inflammatory-related pathways and reduce proinflammatory markers [118,119,120,121,122]. Additionally, amentoflavone isolated from methanolic extracts of *J. communis* leaves was shown to be useful in controlling inflammation, by reducing joints’ rigidity and increasing locomotion after 35 days of treatment in rats with Freund’s adjuvant-induced arthritis at a dose of 40 mg/kg [123].

**Table 4:** In vitro and in vivo health benefits of *Juniperus communis* extracts.

Part of the Plant	Origin	Extract	Subspecies/ Variety	Experimental Model	Effect	References
**Anti-inflammatory and antinociceptive properties**						
**In vitro assay**						
Plant parts	Sweden	Aqueous	n.s.	Prostaglandin biosynthesis assay Platelet activating factor-induced exocytosis assay	↓↓ prostaglandins by 55% at 200 µg/mL ↓↓ platelet activating factor-induced exocytosis by 78% at 250 µg/mL	[25]
Woods	Austria	Methylene chloride	n.s.	12(S)-lipoxygenase assay	↓↓ 12[S]-hydroxy-5,8,10,14-eicosatetraenoic acid by 54.0% at 100 µg/mL, 66.2 and 76.2%,	[88]
Berries	Austria	Methylene chloride	n.s.		↓↓ 12[S]-hydroxy-5,8,10,14-eicosatetraenoic acid by 66.2% at 100 µg/mL	
		Ethyl acetate			↓↓ 12[S]-hydroxy-5,8,10,14-eicosatetraenoic acid by 76.2% at 100 µg/mL	
Plant material (twigs, leaves, and fruits)	Spain	Essential oil	n.s.	Inhibition of nitric oxide production in lipopolysaccharide-activated murine macrophage RAW 264.7 cells	IC_50_ values from 23.98 to 84.80 µg/mL	[75]
**In vivo assays**						
Berries	Italia	Hydroethanolic (80% ethanol, *v/v*)	var. *communis*	Effects on the inhibition of writhing carrageenin foot edema in male Wistar rats after 7 days of treatment at doses of 100 and 200 mg/kg	↓↓ carrageenin-foot edema by 60% and 79% at 100 and 200 mg/kg, respectively	[113]
	Turkey	Aqueous			12.8% inhibition (berries)	[114]
Berries, leaves, and stems	Turkey	Methanolic	var. *communis*		18.5% inhibition (stems) 3.9% inhibition (berries) 18.5% inhibition (leaves)	
		Aqueous	var. *saxatilis*		9.1% inhibition (berries) 7.8% inhibition (leaves)	
		Methanolic			30.5% inhibition (berries) 35.2% inhibition (leaves)	
		Aqueous	var. *communis*	Effects on stimulating response latency in male Swiss albino mice using a hot plate after administration of 100 mg/kg of extract	4.27% inhibition (stems) 5.36% inhibition (berries) 4.29% inhibition (leaves)	
		Methanolic			4.40% inhibition (stems) 4.11% inhibition (berries) 5.16% inhibition (leaves)	
		Aqueous	var. *saxatilis*		3.26% inhibition (stems) 4.32% inhibition (berries) 5.13% inhibition (leaves)	
		Methanolic			3.13% inhibition (stems) 4.05% inhibition (berries) 5.31% inhibition (leaves)	
		Aqueous	var. *communis*	Effects on carrageenin-induced hind-paw edema in male Swiss albino mice after 360 min of 100 mg/kg extract administration	65.9% inhibition (stems) 65.1% inhibition (berries) 65.4% inhibition (leaves)	
		Methanolic			54.3% inhibition (stems) 65.8% inhibition (berries) 54.8% inhibition (leaves)	
		Aqueous	var. *saxatilis*		69.6% inhibition (stems) 51.9% inhibition (berries) 53.6% inhibition (leaves)	
		Methanolic			65.7% inhibition (stems) 43.4% inhibition (berries) 45.3% inhibition (leaves)	
		Methanolic	var. *saxatilis*	Effects on PGE_2_-induced hind-paw edema effects in male Swiss albino mice after 360 min of 100 mg/kg extract administration	17.6% inhibition (stems) 16.5% inhibition (berries) 16.8% inhibition (leaves)	
Leaves	India	Methanolic	n.s.	In vivo study involving different nociceptive assays (acetic acid-induced writhing, formalin and tail-flick tests) in Swiss albino mice at 100 and 200 mg/kg	↓↓ writhing response and the late phase related with the formalin test Act centrally since the extract and pethidine effects were blocked by naloxone in the tail-flick test	[29]
Berries	Romania	Hydroethanolic microemulsions	n.s.	Effects on paw edema in dextran-induced inflammation Wistar rats’ model	↓↓ paw edema	[40]
Berries	Romania	Hydroethanolic microemulsions	n.s.	Kaolin-induced inflammation in Wistar rats’ model	↓↓ interleukins -1β and 6 expression ↓↓ tumor necrosis factor alfa	
**Antidiabetic, antihypercholesterolemic and antihyperlipidemic effects**						
**In vitro assays**						
Fruits	Turkey	Hydroethanolic (80% ethanol, *v/v*)	var. *saxatilis*	Capacity to inhibit *α-*amylase activity	Inhibitory value of 29.8% at 3 mg/mL	[66]
				Capacity to inhibit *α-*glucosidase activity	IC_50_ value of 4.4 µg/mL	
Leaves	Turkey	Hydroethanolic (80% ethanol, *v/v*)	var. *saxatilis*	Capacity to inhibit the *α-*amylase activity	Inhibitory value of 84.3% at 3/mg/mL	
				Capacity to inhibit the *α-*glucosidase activity	IC_50_ value of 53.6 µg/mL	
Plant material	United Kingdom	Aqueous	n.s.	Effects on glucose movement	↓↓ glucose diffusion by 6% at 50 g/L	[124]
**In vivo assays**						
Berries	United Kingdom		n.s.	Streptozotocin-induced diabetic mice models for 40 days at doses of 1 g/400 mL	↓↓ polydipsia Prevent weight losses	[125]
	Spain	Aqueous	n.s.	Effects on streptozotocin-induced diabetic rat models after 24 days of treatment at doses of 250 and 500 mg/kg	↓↓ hypoglycemia in normoglycemic rats	[126]
				Effects on streptozotocin-induced diabetic rat models after 24 days of treatment at 125 mg/kg	↓↓ blood glucose levels and mortality index Prevent weight losses	
	Turkey	Oil dissolved in 0.5% of sodium carboxymethyl cellulose	n.s.	Effects on albino Wistar rats after 30 days of treatment at doses of 50, 100 and 200 mg/kg	↓↓ total cholesterol, oxidized low-density lipoprotein, alanine aminotransferase, and aspartate transaminase levels ↑↑ blood urea nitrogen and creatinine levels	[33]
Plant	n.s.	Methanolic extracts	n.s.	Effects on streptozotocin-nicotinamide induced diabetic rats after 21 days of treatment at doses of 100 and 200 mg/kg	↓↓ blood glucose levels, total cholesterol, triglycerides, low-density lipoprotein, and very-low-density lipoprotein cholesterols ↑↑ high-density lipoprotein cholesterol	[31]
Herbal preparation also composed of *Juniperus. communis*	Croatia	Hydroethanolic (60% ethanol, *v/v*)	n.s.	Effects on alloxan-induced nonobese diabetic NOD mice after 7 days of treatment at 20 mg/kg	↓↓ glucose and fructosamine levels	[127]
**Antiproliferative effects**						
**In vitro assays**						
Berries	Nepal	Aqueous	n.s.	Effects on OECM-1 human gingival squamous cancer cells after 24 h of exposure	Induce apoptosis, exhibiting an IC_50_ value of 46.20 µg/mL	[128]
Plant material	n.s	Aqueous	n.s	Effects on CE81T/VGH human esophageal squamous cell carcinoma after 24, 48 and 72 h of exposure	Induce cell cycle arrest at the G0/G1 phase by regulating the expression of p53/p21 and CDKs/cyclins, triggering cell apoptosis by activating both the extrinsic (Fas/FasL/Caspase 8) and intrinsic (Bcl-2/Bax/Caspase 9) apoptosis pathways IC_50_ values of 68.41, 64.33, and 60.07 µg/mL after 24, 48, and 72 h of exposure, respectively	[39]
				Effects on CE48T/VGH human esophageal epidermoid carcinoma after 24, 48, and 72 h of exposure	Induce cell-cycle arrest at the G0/G1 phase, by regulating the expression of p53/p21 and CDKs/cyclins, triggering cell apoptosis by activating both the extrinsic (Fas/FasL/Caspase 8) and intrinsic (Bcl-2/Bax/Caspase 9) apoptosis pathways IC_50_ values of 69.38, 56.96, and 36.10 µg/mL after 24, 48, and 72 h of exposure, respectively	
	USA	Distilled extracts		Effects on B16/F10 melanoma cells after 24 and 48 h of exposure	Induced apoptosis, decreased angiogenesis and metastasis, and diminished cancer stem-cell expression IC_50_ values of 27 and 44 µg/mL, after 24 and 48 h of exposure, respectively	[73]
Leaves	Turkey	Methanolic	n.s.	Effects on C6 rat brain tumor and HeLa human cervix carcinoma cells after 24 h of exposure	IC_50_ value of 28.43 µg/mL (C6 rat brain tumor) IC_50_ value of 32.96 µg/mL (HeLa cancer cells)	[72]
Aerial parts	Egypt	Methanolic	n.s.	Effects on PC3 human prostate, HCT 116 human colon, and MCF7 breast cancer cells after 24 h of exposure	IC_50_ value of 23.8 µg/mL (PC3 cancer cells) IC_50_ value of 37.6 µg/mL (HCT 116 cancer cells) IC_50_ value of 23.8 µg/mL (MCF7 cancer cells)	[129]
Plant material	New Mexico, USA	Aqueous	n.s.	Effects on MCF-7/AZ breast cancer cells after 24 h of exposure	IC_50_ value of 50 µg/mL	[75,130]
	Spain	Essential oil	n.s.	Effects on NCI-H460 lung, MCF-7 breast, AGS gastric, and Caco-2 cancer cells after 24 h of exposure	IC_50_ values varying from 41.99 to 44.87 µg/mL (NCI-H460 cancer cells) IC_50_ values varying from 30.88 to 163.99 µg/mL (MCF-7 cancer cells) IC_50_ values varying from 132.68 to 302.86 µg/mL (AGS cancer cells) IC_50_ values varying from 107.65 to 230.79 µg/mL (Caco-2 cancer cells)	
Berries	Australia	Methanolic	n.s.	Effects on Caco-2 human colorectal and HeLa cervical cancer cells after 12 h of exposure	IC_50_ value of 1383 µg/mL (Caco-2 cancer cells) IC_50_ value of 2592 µg/mL (HeLa cancer cells)	[131]
		Aqueous	n.s.	Effects on Caco-2 human colorectal and HeLa cervical cancer cells after 12 h of exposure	IC_50_ value of 1516 µg/mL (Caco-2 cancer cells) IC_50_ value of 2157 µg/mL (HeLa cancer cells)	
	Serbia	Essential oil and Distilled extracts	var. *saxatilis*	Effects on A549 human lung adenocarcinoma epithelial cells after 24 h of treatment after 24 h of exposure	Induced apoptosis and arrested cell cycle in G2/M IC_50_ value of 69.4 µg/mL (essential oil) IC_50_ value 1270 µg/mL (distilled extract)	[71]
	USA	Distilled extracts	n.s.	Effects on HepG2 human hepatocellular cancer cells after 24, 48, and 72 h of exposure	IC_50_ values of 48.9, 42.3, and 43.9 µg/mL, after 24, 48, and 72 h of exposure, respectively	[132,133]
				Effects on Mahlavu human hepatocellular carcinoma cells after 24, 48, and 72 h of exposure	IC_50_ values of 64.9, 58.5, and 59.4 µg/mL, after 24, 48, and 72 h of exposure, respectively	
				Effects on J5 human hepatocellular carcinoma cells after 24, 48, and 72 h of exposure	IC_50_ values of 74.2, 67.2, and 53.2 µg/mL, after 24, 48, and 72 h of exposure, respectively	
				Effects on HT-29 colon cancer cells after 24, 48, and 72 h of exposure	Induced cell-cycle arrest at the G0/G1 phase via regulation of p53/p21 and CDK4/cyclin D1 Induced cell apoptosis via the extrinsic (FasL/Fas/caspase-8) and intrinsic (Bax/Bcl-2/caspase-9) apoptotic pathways IC_50_ values of 66.71, 60.02, and 54.32 µg/mL, after 24, 48, and 72 h of exposure, respectively	
				Effects on CT-26 colon cancer cells after 24, 48, and 72 h of exposure	Induced cell-cycle arrest at the G0/G1 phase via regulation of p53/p21 and CDK4/cyclin D1 Induced cell apoptosis via the extrinsic (FasL/Fas/caspase-8) and intrinsic (Bax/Bcl-2/caspase-9) apoptotic pathways IC_50_ values of 27.8, 22.7, and 27.3 µg/mL, after 24, 48, and 72 h of exposure, respectively	
Leaves and branches	Wyoming, USA	Essential oil	n.s.	Effects on SH-SY5Y human neuroblastoma cells after 24 h of exposure	IC_50_ value of 53.7 µg/mL	[134]
Seed cones	Serbia	Essential oil	var. *saxatilis*	Effects on HT-29 and HCT116 colon cancer cells after 24 h of exposure	IC_50_ value 125 µg/mL (HT-29) IC_50_ value of 62.5 µg/mL (HCT116)	[93]
		Distilled extracts			IC_50_ value 625 µg/mL (HT-29) IC_50_ value of 1250 µg/mL (HCT116)	
Roots	China	Acetone	n.s.	Effects on N18 neuroblastoma cell lines after 24 and 48 h of exposure	Induced glioma cell-cycle arrest through intrinsic and extrinsic apoptotic pathways IC_50_ values of 61.11 and 68.94 µg/mL, after 24 and 48 h of exposure, respectively	[135]
				Effects on DBTRG-05MG, G5T/VGH, GBM8401, GBM8901, and RG2 glioblastoma cell lines after 24 h of exposure	Induced glioma cell-cycle arrest through intrinsic and extrinsic apoptotic pathways IC_50_ value of 67.04 µg/mL (DBTRG-05MG glioblastoma cells) IC_50_ value of 63.3 µg/mL (G5T/VGH glioblastoma cells) IC_50_ value of 57.14 µg/mL (GBM8401glioblastoma cells) IC_50_ value of 58.45 µg/mL (GBM8901 glioblastoma cells) IC_50_ value of 69.97 µg/mL (RG2 glioblastoma cells)	
				Effects on DBTRG-05MG, G5T/VGH, GBM8401, GBM8901, and RG2 glioblastoma cell lines after 48 h of exposure	Induced glioma cell-cycle arrest through intrinsic and extrinsic apoptotic pathways IC_50_ value of 49.46 µg/mL (DBTRG-05MG glioblastoma cells) IC_50_ value of 67.85 µg/mL (G5T/VGH glioblastoma cells) IC_50_ value of 46.68 µg/mL (GBM8401glioblastoma cells) IC_50_ value of 55.49 µg/mL (GBM8901 glioblastoma cells) IC_50_ value of 53.8 µg/mL (RG2 glioblastoma cells)	
**In vivo assays**						
Plant	USA	Distilled extracts	n.s.	Effects on melanoma tumor model in C57BL/6 mice after 23 days of treatment at a dose of 200 mg/kg	Cell-cycle arrest at the G0/G1 phase ↓↓ tumor size by 45.2%, B-cell lymphoma-2 (Bcl-2), procaspases 8 and 9 and higher levels of Bcl-2-associated X protein, apoptosis-inducing factor, cell-surface death receptor Fas and Fas ligand when compared to untreated control	[73]
Berries	USA	Distilled extracts	n.s.	Effects in BALB/c nude mice injected with HepG2 liver cancer cells at a dose of 200 mg/kg	↓↓ tumor size ↑↑ lifespan with no or low systemic and pathological toxicity	[132]
				Effects in female BALB/c mice injected with CT-16 colon cancer cells at a dose of 200 mg/kg	Inhibited proliferation Induced apoptosis No obvious change in body weight or histological morphology of normal organs after treatment	[133]
Roots	China	Acetone	n.s.	Effects in male Foxn1 nu/nu mice injected with DBTRG-05MG human glioblastoma cells after 100 days of treatment at a dose of 200 mg/kg	Can penetrate the blood-brain barrier ↓↓ tumor size and the degree of neovascularization ↑↑ PCNA, VEGFR-1, and VEGFR-2 in 44.49%, 5.88%, and 5.85%, respectively, when compared to untreated control	[135]
**Neuronal effects and anticataleptic activity**						
**In vitro assays**						
Leaves	Turkey	Hydroethanolic (80% ethanol, *v/v*)	var. *alpina*	Capacity to inhibit acetylcholinesterase activity	10.38% inhibition at 50 µg/mL 24.30% inhibition at 100 µg/mL 32.89% inhibition at 200 µg/mL	[61,107]
Ripe Berries		Aqueous			5.47% inhibition at 100 µg/mL 28.17% inhibition at 200 µg/mL	
Shoots		Ethyl acetate, ethanolic, and acetone extracts	n.s.		21.34% inhibition at 100 µg/mL (ethyl acetate extract) 13.46% inhibition at 100 µg/mL (ethanolic extract) 28.43% inhibition at 100 µg/mL (acetone extract)	
					Inhibitory percentages varying from 32.34 to 41.97%% inhibition at 100 µg/mL (ethyl acetate extract) Inhibitory percentages varying from 22.29 to 45.45% inhibition at 100 µg/mL (ethanolic extract) Inhibitory percentages varying from 1.91 to 38.55% inhibition at 100 µg/mL (acetone extract)	
Leaves		Ethyl acetate, ethanolic, and acetone extracts	n.s.		20.02% inhibition at 100 µg/mL (ethyl acetate extract) 10.56% inhibition at 100 µg/mL (ethanolic extract) 32.34% inhibition at 100 µg/mL (acetone extract)	
Ripe berries and leaves	Turkey	Aqueous	var. *alpina*	Capacity to inhibit butyrylcholinesterase activity	25.87 (berries) and 25.33% (leaves) inhibition at 50 µg/mL 32.57 (berries) and 44.16% (leaves) inhibition at 100 µg/mL 36.97 (berries) and 62.01% (leaves) inhibition at 200 µg/mL	[61,107]
		Hydroethanolic (80% ethanol, *v/v*)			43.68 (berries) and 30.31% (leaves) inhibition at 50 µg/mL 45.19 (berries) and 33.17% (leaves) inhibition at 100 µg/mL 47.55 (berries) and 35.33% (leaves) inhibition at 200 µg/mL	
Unripe berries		Hydroethanolic (80% ethanol, *v/v*)			44.17% inhibition at 50 µg/mL 48.96% inhibition at 100 µg/mL 49.95% inhibition at 200 µg/mL	
**In vivo assays**						
Leaves	n.s.	Methanolic	n.s.	Effects on Wistar rats with induced Parkinson’s disease by chlorpromazine for 21 days at a dose of 200 mg/kg	↑↑ locomotor activity ↓↓ motor dysfunctions, including catalepsy and muscle rigidity	[19]
Plant material	India	Methanolic		Effects on Wistar rats with induced catalepsy by reserpine 4 h after juniper treatment at a dose of 200 mg/kg	↓↓ catalepsy activity	[34]
	Romania	Essential oil		Effects of juniper volatile oil (1% and 3%) daily inhalation on Amyloid Beta (1–42) male Wistar rat model of Alzheimer’s disease after 21 days of treatment	↑↑ working memory and reference memory errors within radial arm maze task ↓↓ spontaneous alternations percentage within Y-maze task	[136]
				Effects of juniper volatile oil (1% and 3%) daily inhalation on Amyloid Beta (1–42)-induced oxidative stress in Wistar rats	↑↑ acetylcholinesterase, superoxide dismutase and catalase activities, and malondialdehyde and protein carbonyl levels ↓↓ glutathione peroxidase-specific activity and the total content of the reduced glutathione	[85]
**Hepatoprotective effects**						
**In vivo assays**						
Leaves	India	Ethyl acetate	n.s.	Effects on Wistar albino rats with hepatic damage caused by paracetamol for 14 days at a dose of 200 mg/kg	↓↓ alkaline phosphatase (−57.41%), direct bilirubin (−30.33%) and total bilirubin (−38.41%), serum alanine aminotransferase (−34.17%), and serum aspartate aminotransferase (−27.58%) when compared to the untreated group Hepatoprotective effects with rearrangement promotion of portal triads and central veins	[65]
Stems	n.s.	Petroleum ether, chloroform, and ethanol extracts	n.s.	Effects on rats with hepatic damage caused by carbon tetrachloride	Hepatoprotective activity	[30]
Co-combination of berries from juniper and *Solanum xanthocarpum*	India	Ethanolic	n.s.	Effects on Wistar albino rats with liver toxicity induced by paracetamol and azithromycin for 14 days at a dose of 200 mg/kg	↓↓ serum glutamate oxaloacetate transaminase (−65.4%), serum glutamate pyruvate transaminase (−59.3%), alkaline phosphatase (66.8%), total bilirubin (62.1%), and liver inflammation Promoting liver tissue’s normal architecture	[3]
**Tyrosinase inhibitory activity**						
**In vitro assays**						
Berries	Republic of Korea	Methanolic	n.s.	Capacity to inhibit tyrosinase activity	about 50% inhibition at 100 µg/mL	[137]
**Renal effects**						
**In vivo assay**						
Berries	Croatia	Aqueous	n.s.	Daily intake of 10% aqueous infusion, 0.1% of oil (with 0.2% Tween 20 solubilizer) by healthy female Wistar rats	↑↑ diuresis and urine excretion without loss of electrolytes	[86]
**Antiurolithiasis effects**						
**In vitro assay**						
Berries	Iran	Hydroethanolic (50% ethanol, *v/v*)	n.s.	Capacity to dissolve urinary stone brought out from human kidney at concentrations of 500, 1000, and 2000 µg/mL	Dissolve urinary stones ↓↓ dry powder weight of stones ↑↑ the ratio of calcium oxalate in normal saline aqueous solution plus stone	[138]
**Gastrointestinal effects**						
**In vivo assays**						
Leaves	India	Methanolic (80% methanol, *v/v*)	n.s.	Effects on adult male Wistar albino rats with ulcers induced by aspirin, serotonin, indomethacin, alcohol, and stress at doses of 50 and 100 mg/kg	↓↓ aspirin, serotonin, indomethacin, alcohol, and stress-induced gastric ulcerations in rats ↑↑ healing rate of acetic acid-induced ulcers in rats	[87]
			n.s.	Effects on pigs with histamine-induced duodenal lesions at doses of 50 and 100 mg/kg	↓↓ histamine-induced duodenal lesions in pigs	
**Vessels and trachea protective effects in passive smoking**						
**In vitro assays**						
Berries	Romania	Aerosols	n.s.	Effects of 3-week juniper aerosols (40 min/day) on female Sprague-Dawley rats firstly exposed to daily passive smoking for 6 weeks	↓↓ acetylcholine endothelial-dependent relaxation	[139]
		Oil	n.s.	Effects of 3-week juniper nebulization (20 min/day) on the respiratory tract of rats which firstly exposed to 2 cigarettes per day, 5 days a week for 6 weeks	Bronchodilator effects mediated by nitric oxide	[140]
**Genotoxicity protective effects**						
**In vitro assays**						
Berries	Romania	Hydroethanolic (50% ethanol, *v/v*)	n.s.	Capacity to exhibit genoprotective effects against aberrations and abnormalities induced by ethanol on root-tip cells of *Allium cepa* L.	Can effectively protect chromosomes aberrations	[40]

n.s.: not specified; IC50: half-maximal inhibitory concentration ↑↑: increase; ↓↓: reduction.

### 4.4. Antidiabetic, Antihypercholesterolemic, and Antihyperlipidemic Effects

#### 4.4.1. In Vitro Studies

Hydroethanolic extracts of *J. communis* leaves and fruits already displayed, through in vitro assays, the ability to inhibit *α-*amylase (inhibitory scores of 29.8 (fruit) and 53.6% (leaf) at 3 mg/mL), and *α*-glucosidase (IC_50_ values of 4.4 and 84.3 µg/mL for fruit and leaf respectively) activities [66]. Moreover, the aqueous extracts of this plant at 50 g/L also showed the capacity to significantly decrease glucose diffusion by 6% when compared with the negative control [124].

#### 4.4.2. In Vivo Studies

Concerning in vivo studies, the capacity of *J. communis* berries (at 1 g/400 mL) revealed the capability to avoid polydipsia and weight losses, and in this way retard the development of diabetes in streptozotocin mice, as reported by Swanston-Flatt and colleagues [125]. Furthermore, decoctions of *J. communis* berries orally administrated at doses of 250 and 500 mg/kg showed potential to reduce hypoglycemia in normoglycemic rats, reduce blood glucose levels and mortality index, and prevent weight loss in streptozotocin-diabetic rats after 24 days of treatment at a dose of 125 mg/kg [126]. In addition, Banerjee and colleagues [31] verified that the oral administration of *J. communis* methanolic extracts (100 and 200 mg/kg) can effectively reduce blood glucose levels, total cholesterol, triglycerides, low-density lipoprotein, and very-low-density lipoprotein cholesterols, and increase high-density lipoprotein cholesterol in streptozotocin-nicotinamide-induced diabetic rats in a dose-dependent manner after 21 days of treatment.

Finally, a herbal preparation from Croatia composed of natural plants, including *J. communis,* also revealed the capacity to reduce glucose and fructosamine levels in alloxan-induced nonobese diabetic mice at 20 mg/kg after a 7-day treatment [127].

In addition to the mentioned, Akdogan and collaborators [33] conducted a one-month in vivo trial based on the daily administration of *J. communis* berry oil (dissolved in 0.5% of sodium carboxymethyl cellulose) in albino Wistar rats and verified that this berry showed potential to reduce cholesterol at concentrations of 50, 100, and 200 mg/kg. Particularly, the highest dose significantly increased blood-urea nitrogen and creatinine levels and reduced total cholesterol, oxidized low-density lipoprotein, alanine aminotransferase, and aspartate transaminase levels by 16%, 24%, 8.2%, and 10% when compared to the untreated cholesterol group. No anaemic effects or distinct morphological changes in rat kidneys were observed.

Briefly, these effects are mainly attributed to the capacity of *J. communis* to interfere with carbohydrate enzymes, increase peripheral glucose consumption, and protect pancreatic *β*-cells from damage [66,126].

### 4.5. Antiproliferative Effects

Considering the crescent incidence of cancer, it is not surprising that several different efforts are being conducted to discover new approaches and alternatives useful to reduce the development and/or to act as a complementary treatment against this malignancy [141]. Among plants, *J. communis* species have been intensively studied [71,73].

#### 4.5.1. In Vitro Studies

Until now, this plant has already shown the in vitro capacity to suppress the growth of many cancer cells. For example, Lee and colleagues [128] revealed that berry extracts can induce apoptosis on OECM-1 human gingival squamous cancer cells, exhibiting an IC_50_ value of 46.20 µg/mL after 24 h of exposure. Furthermore, it was also reported that this plant can interfere with the growth of CE81T/VGH human esophageal squamous carcinoma (IC_50_ scores of 68.41, 64.33, and 60.07 µg/mL after 24, 48, and 72 h of exposure, respectively) and CE48T/VGH human esophageal epidermoid carcinoma cells (IC_50_ values of 69.38, 56.96, and 36.10 µg/mL after 24, 48, and 72 h of exposure, respectively), mainly by inducing cell-cycle arrest at the G0/G1 phase [39]. Methanolic extracts of its leaves also showed capacity to block the growth and development of C6 rat-brain tumor and HeLa human-cervix carcinoma cells (IC_50_ values of 28.43 and 32.96 µg/mL, respectively) [72], PC3 human-prostate cancer cells (IC_50_ = 23.8 µg/mL), HCT 116 human-colon cancer cells (IC_50_ = 37.6 µg/mL), and MCF7 breast cancer cells (IC_50_ = 23.8 µg/mL) after 24 h of exposure [129]. On the other hand, aqueous berry extracts can decrease the growth and invasion of MCF-7/AZ breast cancer cells (IC_50_ value of 50 µg/mL after 24 h of treatment) [130]. In addition to the mentioned, Fernandez and coworkers [131] also reported that methanolic extracts of its berries can block the proliferation of Caco-2 human colorectal and HeLa cervical cancer cells, showing IC_50_ values of 1383 and 2592 µg/mL, respectively, after 12 h of exposure. Their aqueous extracts also showed potential to inhibit both cancer cells after 12 h, exhibiting IC_50_ scores of 1516 (Caco-2 cancer cells) and 2157 µg/mL (HeLa cancer cells) [131]. On the other hand, essential oil and distilled extracts from *J. communis* berries revealed the potential to suppress A549 human lung adenocarcinoma epithelial-cell growth and development, revealing IC_50_ values of 69.4 and 1270 µg/mL, respectively, after 24 h of treatment [71]. Additionally, they also showed the ability to suppress the development of SH-SY5Y human neuroblastoma cells after 24 h of exposure (IC_50_ score of 53.7 µg/mL), which is evidence that this plant can penetrate the blood–brain barrier [134,135,142]. The capacity of *J. communis* plant material (twigs, leaves and berries) to suppress NCI-H460 lung carcinoma, MCF-7, AGS gastric carcinoma, and Caco-2 cell growth was also evaluated, revealing IC_50_ values varying depending on the origin [75]. Essential oil and distilled extracts of seed cones from *J. communis* also reveal the capacity to inhibit the growth of HT-29 (IC_50_ values of 125 and 625 µg/mL for essential oil and distilled extracts, respectively) and HCT116 cancer cells (IC_50_ values of 62.5 and 1250 µg/mL for essential oil and distilled extracts, respectively) after 24 h of exposure [93].

*J. communis* distilled extracts also seem to be useful in the prevention of melanoma tumorigenesis, since they already show potential to block B16/F10 melanoma cells growth, displaying IC_50_ values of 27 and 44 µg/mL after 24 and 48 h of exposure, respectively [73]. These data are in agreement with in vivo results [73]. Furthermore, this plant also showed potential to inhibit HepG2, Mahlavu, and J5 human hepatocellular carcinoma cell growth, in a dose- and time-dependent manner, revealing IC_50_ values of 43.9 µg/mL for HepG2 cells, 59.4 µg/mL for Mahlavu cells, and 53.2 µg/mL for J5 cells after 72 h of treatment [132].

#### 4.5.2. In Vivo Studies

The administration of *J. communis* distilled extracts (200 mg/kg) for 23 days C57BL/6 mice showed the capacity to reduce tumor size by 45.2% when compared to the untreated group. It was also verified that *J. communis* treatment resulted in cell-cycle arrest at the G0/G1 phase; lower concentrations of B-cell lymphoma-2 (Bcl-2), procaspases 8 and 9; and higher levels of Bcl-2-associated X protein, apoptosis-inducing factor, cell-surface death receptor Fas, and Fas ligand [73]. On the other hand, the administration of *J. communis* essential oils (200 mg/kg) in BALB/c nude mice injected with HepG2 cancer cells showed the capacity to reduce tumor growth and extend the lifespan with no or low systemic and pathological toxicity [132]. Similar information was reported by Lai and colleagues [133] and Tsai and collaborators [135] regarding the antitumor effects of this plant against human colorectal adenocarcinoma and glioblastoma.

Remarkably, the antiproliferative and cytotoxic effects shown by this plant are mainly attributed to the capacity of phenolic compounds and terpenes to interact, in different ways, with cell-signaling pathways and cascades, inducing apoptosis and interfering with cell cycle progression [71,73,132,142]. Particularly, imbricatolic acid isolated from the methanolic extract of *J. communis* fresh ripe berries showed the ability to prevent cell-cycle progression in p53-null human lung cancer Calu-6 cells by inducing the upregulation of cyclin-dependent kinase inhibitors and their accumulation in the G1 phase of the cell cycle, as well as the degradation of cyclins A, D1, and E1 [143]. On the other hand, isocupressic acid and deoxypodophyllotoxin isolated from this plant can induce caspase-dependent apoptosis in malignant MB231 breast cancer cells; additionally, deoxypodophyllotoxin also showed the potential to inhibit cell-survival pathways mediated by MAPK/ERK and NF*κ*B-signaling pathways [144].

### 4.6. Neuronal Effects and Anticataleptic Activity

*J. communis* parts also show great potential to working memory, and inhibit the activity of some enzymes associated with the progression of neurological pathologies, such as Alzheimer’s and Parkinson’s disease [19,61,85].

#### 4.6.1. In Vitro Studies

Focusing on in vitro assays, acetylcholinesterase inhibitory percentages ranging from 5.47% (leaf hydroethanolic extracts at concentration of 100 µg/mL) to 32.89% (berries aqueous extracts at 200 µg/mL) were reported. Additionally, and regarding the inhibition of butyrylcholinesterase, scores ranging between 25.33% and 62.01% for leaf aqueous extracts at concentrations of 50 and 200 µg/mL were reported, and from 25.87% to 49.95% regarding aqueous extracts of its ripe berries at the same concentrations mentioned above [61]. Furthermore, ethyl acetate and ethanolic extracts of its shoots revealed the ability to inhibit both enzymes at a concentration of 100 µg/mL (inhibitory percentages of 20.02 and 21.34% for ethyl acetate extract regarding acetylcholinesterase and butyrylcholinesterase inhibition, respectively, and 22.29 and 45.45% for acetylcholinesterase and butyrylcholinesterase inhibition for the ethanolic extract, respectively) [107].

#### 4.6.2. In Vivo Studies

Regarding in vivo assays, Bais et al. [19] reported that the daily administration of 200 mg/kg (i.p.) of *J. communis* methanolic extracts for 21 days in rats with induced Parkinson’s disease by chlorpromazine can effectively decrease motor dysfunctions, including catalepsy and muscle rigidity, and increase locomotor activity when compared to the untreated group. The obtained results are in line with previous data, which showed that the daily injection of similar extract (200 mg/kg, i.p.) can significantly reduce the retention on bar (catalepsy activity) by 75% in rats with induced catalepsy by reserpine [34]. In addition to the mentioned, the daily inhalation of 1% and 3% for 60 min during 21 days of juniper volatile oils extracted from *J. communis*, mainly composed of *α*-pinene, sabinene, myrcene, limonene, terpinen-4-ol, and *α*-thujene, by rats with induced Alzheimer’s disease, showed increases in working and long-term memories and decreases in acetylcholinesterase activity [85,136].

### 4.7. Hepatoprotective Effects

#### In Vivo Studies

Ethyl acetate fractions of leaves from *J. communis* have already been shown to be promising hepatoprotective agents. Rats with hepatic damage caused by paracetamol who ingested these fractions (200 mg/kg body weight) over two weeks showed lower levels of alkaline phosphatase (−57.41%), direct bilirubin (−30.33%) and total bilirubin (−38.41%), serum alanine aminotransferase (−34.17%), and serum aspartate aminotransferase (−27.58%) than the untreated group. Histopathological observations also proved the hepatoprotective effects of these leaves, promoting favorable portal triads and central-vein rearrangements [65]. Using a carbon tetrachloride-induced hepatic damage model, Mavin and Garg [30] revealed similar effects of *J. communis* stems. Furthermore, Singh et al. [145] reported that the daily ingestion of a combination of ethanolic berry extract of *Solanum xanthocarpum* (200 mg/kg) and *J. communis* (200 mg/kg) for 14 days can significantly attenuate liver toxicity induced by paracetamol and azithromycin in Wistar albino rats. In fact, the administration of both showed a capability to reduce altered biochemical parameters, including serum glutamate oxaloacetate transaminase (−65.4%), serum glutamate pyruvate transaminase (−59.3%), alkaline phosphatase (66.8%), and total bilirubin (62.1%), and reverse histopathological alterations, by promoting the liver tissue’s normal architecture and diminishing liver inflammation.

### 4.8. Tyrosinase Inhibitory Activity

#### In Vitro Studies

Methanolic extracts of berries from *J. communis* already showed the capacity to suppress mushroom tyrosine activity by about 50% at concentrations of 100 µg/mL. This data is very promising and can be considered an indicator regarding the potential of this plant to treat skin disorders, since this enzyme is closely involved in the production of melanin. Moreover, some compounds isolated from them also showed similar potential, namely hypolaetin 7-*O-β*-xylopyranoside, which exhibited an IC_50_ value of 45.15 µM, and kojic acid (IC_50_ score of 25.03 µM) [137].

### 4.9. Renal and Antiurolithiasis Effects

#### 4.9.1. In Vitro Studies

Relative to antiurolithiasis properties, *J. communis* berries at concentrations of 500, 1000, and 2000 µg/mL solutions showed potential to dissolve urinary stones brought out from the human kidney, causing reductions of 50, 20, 10, and 20% in urinary stones composed of calcium oxalate, calcium hydrogen phosphate, magnesium ammonium phosphate, and ammonium urate, respectively. The dry-powder weight of stones in normal saline also decreased from 1458 to 1162, 1124, 1136, 1144, 1096, 1126, and 1130 mg after exposure to increasing *Juniperus* berry concentrations. Furthermore, it was also observed that the ratio of calcium oxalate in normal saline aqueous solution plus stone increased from 70% to 80% after using some fractions of *J. communis* berry extracts [138].

#### 4.9.2. In Vivo Studies

Different parts of *J. communis* plants have been largely used since ancient times to treat renal disorders because of their diuretic and urinary antiseptic effects. Indeed, Stanic et al. [86] reported that the daily intake of 10% aqueous infusion, 0.1% of oil (with 0.2% Tween 20 solubilizer) from juniper berries, and 0.01% of terpinen-4-ol (one of the main components of *Juniperus* plants) in rats at 5 mL/100 g can effectively stimulate diuresis from day 2, increasing urine excretion without loss of electrolytes. Between them, the infusion showed the most prominent diuretic activity (+43% on day two and 44% on day three), which proves that the diuretic activity of juniper berries is due to the combination of essential oil and hydrophilic components, which together can increase the glomerular filtration rate. Even so, recent studies use do not recommend their continuous use due to the presence of terpinen-4-ol, which has already been shown to promote kidney irritation [146].

### 4.10. Gastrointestinal Effects

#### In Vivo Studies

Pramanik and colleagues [87] reported that *J. communis* leaves can be useful in ameliorating some gastrointestinal ailments. The authors verified that the intraperitoneal administration of the methanolic extract at doses of 50 and 100 mg/kg can effectively inhibit aspirin, serotonin, indomethacin, alcohol, and stress-induced gastric ulcerations in rats, and histamine-induced duodenal lesions in guinea pigs. The treatment with the leaf extract also enhanced the healing rate of acetic acid-induced ulcers in rats. Additionally, the analysis of gastric juice revealed that although the leaf extract did not alter its pH or its peptic activity, this one managed to significantly diminish its volume and total acidity. These benefits shown by *J. communis* parts are positively linked to their anti-inflammatory and analgesic properties.

### 4.11. Vessels and Trachea Protective Effects in Passive Smoking

#### In Vivo Studies

The capacity of *J. communis* aerosols to reverse the vasomotor impairment associated with passive exposure to cigarette smoke was also evaluated in female Sprague Dawley rats. Animals were first exposed to daily passive smoking for 6 weeks. In the last 15 days of the study, one of the groups was also subject to a daily administration of *J. communis* oil aerosols for 40 min/day. In the end, thoracic aortas were harvested and analyzed, and it was possible to verify that the use of aerosols can significantly reduce acetylcholine endothelial-dependent relaxation [139]. Furthermore, Pleşa and colleagues [140] reported that the nebulization with *J. communis* berry oil (20 min/day per 3 weeks) exerts bronchodilator effects mediated by nitric oxide in the respiratory tract of rats exposed to 2 cigarettes per day, 5 days a week for 6 weeks. This activity is closely linked to the antioxidant effects shown by this plant. Even so, the authors also verified that this aerosol exposure can cause moderate irritation and inflammation along the tracheobronchial tract in nonsmoker rats.

### 4.12. Genotoxicity Protective Effects

#### In Vitro Studies

Recently, *J. communis* berries displayed capacity to inhibit chromosome aberrations and mitotic abnormalities induced by ethanol on *Allium cepa* L. root-tip cells, with these properties being intimately linked to their capacity to scavenge radicals and reduce oxidative stress levels [40].

### 4.13. Toxicity Effects

#### 4.13.1. In Vitro Acute Toxicity

Fernandez and colleagues [131] assessed the toxicity of methanolic and aqueous berry extracts of *J. communis* through *Artemia franciscana* nauplii lethality assay, and proved their safety once the obtained IC_50_ values were higher than 1 mg/mL. Additionally, the toxicity and undesirable side effects of *J. communis* were evaluated in albino rats based on the oral administration of ethyl acetate fractions of their leaves for 2 weeks. The obtained data revealed no mortality nor any negative change in physiological parameters and appearance until the dose of 2 g/kg [65].

#### 4.13.2. Antiprogestogenic and Abortifacient In Vivo Effects

Pathak and colleagues [147] reported that hydroethanolic extracts (90% ethanol, *v/v*) of *J. communis* berries did not show estrogenic nor antiestrogenic effects, but displayed antiprogestational and antifertility activity at doses ranging from 50–450 mg/kg on female rats. In another study, Agrawal et al. [148] found that the oral administration of hydroethanolic extracts (50% ethanol, *v/v*) from *J. communis* berries at doses of 300 and 500 mg/kg in albino female rats from day 1 to day 7 of pregnancy exhibited dose-dependent anti-implantation activity. Furthermore, the authors also reported that these extracts at the same concentrations promoted abortifacient effects when administrated on days 14, 15, and 16 of pregnancy. Still, no evidence of teratogenicity effects was found.

## 5. Conclusions

The pharmacological effects of the zimbro plant have been known since ancient times, and are mainly attributed to the high concentration of phenolic compounds, in particular the presence of 5-*O*-caffeoylquinic and quinic acids, catechin, epicatechin, amentoflavone, quercetin, luteolin, apigenin, and naringenin; and VOCs, namely monoterpenes and sesquiterpenoids. In fact, these phytochemicals confer remarkable biological activities, such as important antimicrobial capacity, the ability to modulate biofilm formation, as well as notable antioxidant, hepatoprotective, anticancer, anti-inflammatory, antihypercholesterolemic, neuroprotective, and genotoxic effects. Given this, it is not surprising that its use and incorporation in dietary supplements, nutraceuticals, and pharmaceutical drugs is a hot topic among researchers, considering its potential to attenuate—or even treat—several diseases and ailments. However, more studies, namely clinical trials, are needed to fully explore and reveal all the biological potentials and optimal doses of this plant.

## Data Availability

All data are reported in the manuscript.
